# Understanding Health Care Workers’ Attitudes and Preferences Toward Digital Patient Monitoring Platforms: Cross-Country Survey Study

**DOI:** 10.2196/67142

**Published:** 2025-09-23

**Authors:** Costanza Tortù, Chiara Seghieri, Ditila Doracaj, Natalya Usheva, Natalia Giménez-Legarre, Yannis Manios

**Affiliations:** 1 Institute of Management, L’EMbeDS, Sant’Anna School of Advanced Studies, Piazza Martiri della Libertà 33, Pisa, 56127, Italy, 39 050883863; 2 Department of Internal Medicine, University of Medicine of Tirana, Tirana, Albania; 3 Department of Social Medicine and Health Care Organization, Medical University of Varna, Varna, Bulgaria; 4 GENUD (Growth, Exercise, Nutrition and Development) Research Group, University of Zaragoza, Zaragoza, Spain; 5 Institute for Health Research Aragón (IIS Aragón), Zaragoza, Spain; 6 Biomedical Research Centre on Obesity Physiopathology and Nutrition (CIBERObn), Carlos III Health Institute, Madrid, Spain; 7 Aragon Agroalimentary Institute (IA2), University of Zaragoza-CITA, Zaragoza, Spain; 8 Department of Nutrition and Dietetics, School of Health Science and Education, Harokopio University, Athens, Greece

**Keywords:** health care workers, health technology, discrete choice experiments, preferences, survey study

## Abstract

**Background:**

The integration of digital health tools into the routines of health care workers (HCWs) holds potential to enhance health care delivery. In particular, digital tools for patient data monitoring allow HCWs to quickly access patient health information and detect early warning signs of potential issues. However, while interest in these tools, such as telemedicine and mobile health, has rapidly grown in recent years, limited research has explored HCWs’ attitudes toward digital innovations or their feature preferences.

**Objective:**

This study aims to (1) assess HCWs’ attitudes toward digital health tools for patient data monitoring, (2) identify socioeconomic factors influencing these attitudes, (3) determine HCWs’ preferences for features of a novel digital health platform for patient data monitoring, and (4) examine whether HCWs’ baseline attitudes impact their feature preferences.

**Methods:**

This study uses an integrated approach combining item response theory (IRT) and discrete choice experiment to evaluate the attitudes and preferences of HCWs. Data come from a web conjoint survey distributed to an international cohort of HCWs across the following 4 European countries: Spain, Albania, Bulgaria, and Greece.

**Results:**

Survey respondents comprise 260 HCWs from the 4 countries. The findings indicate that HCWs generally hold a positive attitude toward technological devices (all the IRT coefficients are statistically significant with *P*<.05). Socioeconomic characteristics, including factors such as gender (P=0.05), professional role (*P*=0.01), and educational background (*P*=0.01), significantly influence these attitudes. Results show that highly educated female HCWs are those who are mostly inclined to use technologies. In addition, the specific features of a digital health platform for patient data monitoring highly impact HCWs’ willingness to incorporate such a tool into their daily practice (all coefficients related to the attributes’ effects in the models for the discrete choice experiment results are significant (all P=0.01 except the data looking attribute which has P=0.03) . The findings suggest that an ideal digital health platform for patient data monitoring should offer intuitive graphs, comparative statistics against standards, and include patients’ family clinical history. In addition, health workers should receive instructor-led group training to effectively use the platform.

**Conclusions:**

This study shows that health workers generally support the use of digital health tools, which have the potential to improve health care efficiency and patient outcomes through enhanced monitoring and timely interventions. To facilitate adoption, policymakers should strengthen infrastructure, enact supportive legislation, and tailor interventions for groups less inclined to use these tools. Aligning digital health platform features with HCW preferences is crucial, as it directly impacts HCWs’ willingness to integrate these tools into daily routines, ultimately benefiting patients. Future research should examine additional factors influencing HCW adoption and address organizational and infrastructural barriers to optimize implementation of digital health platform and improve patient care.

## Introduction

In recent years, there has been a growing interest in the scientific debate on telemedicine [1-3]. The field of telemedicine has become increasingly prominent, as advancements in technology and communication have opened up new possibilities for delivering health care services remotely [4]. This evolving trend has sparked discussions among researchers, health care professionals, and policymakers about the potential benefits and challenges associated with the widespread adoption of telemedicine [5,6]. The term “telemedicine” refers to the adoption of technological tools to provide medical information and services from a distance. This can include online consultations [7,8], remote monitoring of patients [9,10], exchange of medical data through digital means, and the usage of other digital technologies [11]. The convenience and accessibility offered by telemedicine have garnered attention, particularly in addressing issues of health care accessibility in remote areas [4,12,13].

The integration of telemedicine technologies can not only enhance the efficiency of health care delivery but also empower both patients and health care providers by fostering continuous and proactive health care management. By embracing these tools, health care workers (HCWs) can offer personalized and responsive care, improving the overall patient outcomes and contributing to the broader evolution of health care toward a more patient-centered and technologically advanced scheme [14-16]. In particular, the use of technological tools for remote monitoring of patient data allows HCWs to gather real-time insights into a patient’s health status, facilitating early detection of potential issues and enabling timely interventions [16].

Following the growing interest in the impact of telemedicine and, particularly, of remote monitoring of patients on the quality of health care, there are several contributions that explore HCW’s perspectives about these new tools [17-22]. However, detailed information on the HCW preferences on how to read and monitor patients’ data in a technological platform is still scarce, being mostly gathered from the patient’s perspective [23,24]. Understanding the attitudes of health workers toward technology, particularly their preferences regarding the usage of digital health, such as mobile health (mHealth) to support health care for reading and monitoring patients’ data and feedback, is of paramount importance. Understanding HCWs’ receptiveness toward these tools and their preferences in terms of user interfaces, data visualization, and ease of integration into existing workflows can significantly impact the efficiency and accuracy of patient data interpretation. This basically ensures that technological solutions align seamlessly with their needs, facilitating a smoother transition to telemedicine. On the other hand, identifying potential concerns or barriers HCWs may have regarding the use of digital health tools for patient data monitoring is essential for targeted training and support [21,25].

In summary, exploring health workers’ attitudes toward digital health tools for reading and monitoring patients’ data is a pivotal step in enhancing the overall effectiveness of telemedicine and in encouraging a technologically empowered health care system. Existing contributions in the field of social research on HCWs’ attitudes and preferences toward digital health tools mainly focus on gathering their opinions based on specific experiences with these tools by using different methodologies.

Systematic reviews in this theme have pointed out that there are technical, social, and organizational factors that may influence the adoption of digital health applications by the health care staff [19,20]. About the technical factors, some existing works have explored the digital health literacy of these professionals [26], while others have collected and evaluated their general attitude and opinions on using such digital health technologies [19-21,26-29]. These analyses encompass the social and organizational elements of HCWs’ preferences and perspectives about digital health tools by either using qualitative methodology [18,19,30,31], mixed methods approaches [23,32], quantitative methodologies [33-36] or discrete choice experiments (DCEs) [37-40], which implicitly elicit health workers’ barriers and facilitators toward digital health technologies.

However, recent contributions state that there remains considerable room for improvement in encouraging health workers to embrace such technologies and enhancing the overall effectiveness of telemedicine even in high-income countries [18,20,22,41]. In particular, the ongoing digitalization of health care causes a change in the traditional role of the health care worker, which may come with specific barriers and challenges [18]. Moreover, it would be important to jointly consider these aspects and to assess whether a different attitude toward the mHealth tools impacts the preferences on the usage of such tools. This integrated approach provides a deeper understanding and enhances the capacity to develop tailored interventions that succeed across diverse contexts, ultimately contributing to the optimization of telemedicine practices globally.

This study proposes an integrated approach aimed at evaluating the receptivity of HCWs toward adopting digital health technologies, while also identifying potential barriers or facilitators that may influence their willingness to use such devices for patient data monitoring. The investigation is planned throughout the EU-funded DigiCare4You project, an innovative intersectoral initiative using digital tools for the prevention and management of type 2 diabetes and hypertension. The project spans across 2 high-income European countries, Spain and Greece, as well as middle-income countries, Bulgaria and Albania.

The digital tools developed within the project are designed for both patients with chronic diseases and HCWs. An mHealth system has been developed consisting of a novel mobile app for patients in which they are encouraged to input data related to their clinical parameters, dietary habits, and exercise routines, and a digital health platform that HCWs are asked to introduce in their workday routine to monitor the data of their patients. Before implementing these digital tools, the project partners conducted a comprehensive analysis to gain insights into the attitudes of both patients and HCWs regarding the use of digital technologies and to understand their preferences about these tools, thus laying the groundwork for the successful implementation and acceptance of the proposed digital solutions.

To explore attitudes and preferences of HCWs toward the platform for patient data monitoring, the project partners planned, developed, and disseminated a Web Conjoint Survey (WCS) across all 4 implementation countries. The WCS comprises three key sections: (1) capturing basic sociodemographic characteristics, (2) delving into the HCWs’ baseline attitude toward technologies, and (3) using a DCE [42] to elicit their preferences about a novel digital health platform to be introduced in their workday routine for patients’ data monitoring.

In particular, the second section, which explores the HCWs’ attitude toward technology, includes some statements with respect to which respondents are asked to declare their level of agreement. These statements describe behaviors or beliefs pertaining to the use of technological devices in their daily work routines and to the integration of technologies in the management of lifestyle habits. The responses from HCWs to these statements are analyzed using item response theory (IRT) models [43,44]. These models enable the estimation of their overall latent propensities to incorporate technological devices into their working activities and into the management of lifestyle habits.

In the third section, HCWs undergo a DCE, designed to elicit their preferences about different specific characteristics of a digital health platform for patients’ data monitoring. They are asked to choose their preferred option in a series of pairwise competing scenarios, each comparing 2 platforms for patients’ data monitoring with different attributes. The attributes considered as potential obstacles or facilitators in platform usage are derived from a thorough literature review and targeted focus groups implemented in the 4 implementation countries involving health care professionals and health care policymakers. The selection of competing scenarios is randomized using an appropriate randomization design.

In analyzing the DCE results using conditional regression models, we also include as a covariate in the regression the information about the predicted latent traits toward the usage of technologies (the estimated attitude toward technology) resulting from the IRT analysis. This integrated analytical approach combining IRT and DCE significantly enhances our understanding of HCWs’ inclinations toward the usage of technological tools and provides valuable insights into their preferences on digital tools for patient data monitoring. Furthermore, this method enables an assessment of whether an interaction exists between HCWs’ baseline attitudes toward technology and their preferences for the platform.

Given that the analysis spans an international cohort of HCWs situated in multiple European countries, the results hold the potential for generalization across diverse contexts.

## Methods

### Overview

This study analyzes the data coming from a web conjoint survey administered to a sample of HCWs in Europe. The web survey was implemented through the online software platform Qualtrics Experience Management. The survey was launched in April 2022 and ended in November 2023. To get access to the survey, the respondent required only an anonymous survey link, which could be easily shared via email, social networks, QR codes, or private messages, ensuring a user-friendly and widespread dissemination. Within the DigiCare4You Project, local partners from the 4 implementation countries were in charge of distributing the survey link to HWs within their network, and those who received the link were asked to further circulate it among their peers. By actively encouraging participants to share the survey link within their working networks, we seek to foster a widespread reach, initiating a dynami*c* snowball sampling mechanism [45]. While acknowledging that the resultant sample is nonprobabilistic, this strategic approach plays a pivotal role in attracting a diverse range of respondents and enriching the data with a multitude of perspectives.

The WCS is structured across three main sections: (1) the first section includes demographic details of respondents, encompassing age, employment, and years of education; (2) the second section evaluates the baseline individual attitude toward technology. In particular, the goal is to understand the propensity of the respondents to use technological devices in their working life and in the managing of their lifestyle habits, by looking at their level of agreement with some statements; (3) the third section provides the DCE experiment to elicit the HCWs preferences on the novel platform to monitor the health status of their patients. The survey was implemented in 5 languages, which include the 4 official languages in the 4 implementation countries of the DigiCare4You project plus the English language. The survey was completely anonymous and does not collect personally identifiable information. The complete survey (in English) and the CHERRIES (Checklist for Reporting Results of Internet E-Surveys) checklist are available as Multimedia Appendix 1 and Checklist 1, respectively.

### Item Response Theory

Considering previous evidence pointing out that the usage of technologies in working activities and in daily life may influence the adoption of digital tools by the health workers [20], the second section of the survey explores the HCWs’ baseline attitudes toward technology. Participants were asked to indicate, on a scale ranging from 1-10, their level of agreement with 10 statements. These statements were designed to capture attitudes and beliefs related to the use of technological devices, with 5 statements focusing on working activities and the remaining 5 statements on the integration of technology into lifestyle management. In this scale, a rating of 1 signifies “totally disagree,” while a rating of 10 indicates “totally agree.” The list of statements is presented in Table 1.

**Table 1.** Survey items regarding the health care workers’ attitude toward using technology both in working activities and in managing lifestyle habits.

Item	Statement
Usage of technologies in working activities	
Work 1	“I think that using health technologies (e.g electronic health records, mHealth, telehealth) in my job would enable me to accomplish tasks more quickly.”
Work 2	“I think that using health technologies (e.g electronic health records, mHealth, telehealth) would make it easier to do my job.”
Work 3	“I would find health technologies (e.g electronic health records, mHealth, telehealth) useful in my job.”
Work 4	“I think that using health technologies (e.g electronic health records, mHealth, telehealth) would improve the quality of the work I do.”
Work 5	“I think that using health technologies (e.g electronic health records, mHealth, telehealth) in my job would increase my productivity.”
Usage of technologies in managing lifestyle habits	
Habits1	“I used to / I would like to track some health parameters (e.g hearth rate, Oxygen saturation, quality of sleep) through a technological device.”
Habits 2	“I like to track my lifestyle habits through a mobile app.”
Habits 3	“I like to monitor what i eat and drink through a mobile app.”
Habits 4	“I like to track the statistics regarding some activity parameters (steps taken, stairs climbed, minutes of workout performed) through a technological device.”
Habits 5	“I like to write on a virtual notebook some notes about what I do in a day and how I feel.”

Using their responses to such statements, we intend to quantify the HCWs’ baseline attitude toward technology. In particular, we intend to estimate their latent attitude in using technological tools for working activities and lifestyle habits using IRT models. These models provide a robust framework for estimating an individual’s latent trait, based on their responses to a set of items [44]. This methodology offers valuable insights into abilities or latent characteristics by considering individual responses across a range of assessment items. IRTs are widely used in educational [46,47], psychological [48], and clinical [49,50] measurement. Using an IRT model, we can estimate, through the maximum likelihood approach, the values assumed by the latent trait of each respondent.

In this study, our primary goal was to assess the attitudes of HCWs toward technology usage, distinguishing between their perspectives on professional tasks and leisure pursuits. To achieve this, we analyzed their responses to distinct sets of items tailored to each aspect, with each item rated on a scale from 1 to 10. To accurately capture and interpret these ordinal responses, we used a specialized form of IRT known as the Graded Response Model (GRM) [51,52]. This model is well suited for assessing attitudes expressed through ordered categories, providing a nuanced understanding of the varying degrees of agreement or disagreement with the given statements related to technology use. In this setting, we estimated 2 separate GRMs to predict the individual attitude of health workers to use technological tools in their working activities and in the management of their lifestyle habits. We denoted these predicted traits as ${\hat{\theta }}_{W}$ and ${\hat{\theta }}_{H}$, respectively. In addition, to enhance the robustness of our analysis, we also estimated the overall latent HCWs’ attitude toward technology ${\hat{\theta }}_{WH}$ by applying GRM to all items pertaining to statements on technology usage in both professional tasks and daily routine. Before fitting GRMs, we noted that there were a few missing entries in the responses to the items. Since, upon exploring the data, we did not observe relevant patterns that rule the missingness in the items, we can impute them through Predictive Mean Matching (PMM) [53,54]. All the descriptives related to the missing data in the items and the performance of the PMM algorithm in imputing missing entries are available in the Multimedia Appendix 2. Finally, after estimating the latent traits, we identified the determinants influencing these traits by means of linear regression models with the latent traits ${\hat{\theta }}_{W}$ , ${\hat{\theta }}_{H}$ and ${\hat{\theta }}_{WH}$ as the outcome variables and individual health worker’s characteristics as regressors. The independent variables in these models encompassed the health worker’s gender, the country of residence, the binary variable less than 30 (indicating whether the health worker is less than 30 years old), the binary variable high education (equal to 1 if the health worker has more than 14 years of education), the variable electronic health record (indicating the health worker’s experience with electronic health records), and the binary variable doctor (taking the value 1 for general practitioners and specialized doctors and 0 for the other health workers’ employment positions).

### Discrete Choice Experiments

The third section of the survey provided a DCE [42] to assess the HCW’s preferences on the digital health platform they might use to monitor the health status of their patients. DCEs are widely used in several fields of social sciences [55,56] and health care applications [42] as they are flexible and easy to interpret. They enable researchers to elicit the user preferences on a specific issue without explicitly asking them, as respondents face some pairwise comparisons and choose their preferred option between 2 competing scenarios, whose characteristics (attributes) provide different options (levels).

In this setting, HCWs indicated the preferred platform for patient data monitoring between 2 competing platforms that differed in the characteristics of some of their aspects. These aspects have been identified based on a literature review of studies using DCE in similar contexts. In addition, insights were gathered through focused group discussions involving health workers and health care policymakers across the 4 implementation countries (details about the participants and the output of the focus groups are available in the Multimedia Appendix 3).

The key aspects considered for the web platform are as follows:

1. Data visualization: Determines whether health workers prefer data presented through tables or graphs;
2. Statistical content: Focuses on the type of statistics preferred by health workers (patient-specific statistics or statistics comparing patients to a standard, such as a national average or app users’ average);
3. Additional content: Explores the extra data health workers desire in the platform beyond clinical parameters and lifestyle habits (eg, patients’ family clinical history or emotional status)
4. Platform usage training: Evaluates the preferred type of training for using the platform (group training with an instructor or self-training with tutorials).

Table 2 describes the attributes and levels that have been used in the DCE. In the proposed DCE, respondents are asked to pick their preferred platform for patient data monitoring between two competing platforms, whose main characteristics –summarized by the attributes- provide different options –represented by the levels. The DCE data were analyzed using alternative-specific conditional logit regression models, in which the outcome was HCW’s preference (ie, platform A or platform B).

**Table 2.** Attributes and levels of the Discrete Choice Experiment.

Attributes	Level 1	Level 2
Data looking	Tables	Graphs
Statistical content	Statistics of my patients	Statistics of a given patient against a standard
Additional contents	Patient’s family clinical history	Patients’ emotional status
Training	Group training with instructor	Self-training with a tutorial

### Ethical Considerations

The study was carried out in compliance and accordance with the General Data Protection Regulation (2016/679) and the Italian Legislative Decree No. 196/2003 (“Personal Data Protection Code ").

The data used in the study were collected from a web survey, which is completely anonymous and does not collect personally identifiable information. In particular, a link to a survey was sent to individual email addresses or through a QR code. Responses were submitted directly to a survey software package (not returned via email). The survey included a disclosure letter describing the project and the purpose of the survey, the section of the questionnaire, the length of time of the survey, and that participation and consent were totally voluntary. The questionnaire did not gather any personally identifiable information for any purpose, or a combination of identifiers that might make it more likely to identify an individual. The individual respondents’ responses and data could not be linked to their email. No incentives were offered.

Since the data do not interfere with the rights and freedoms of the respondents, an evaluation by the Ethics Committee is not necessary. According to the Declaration of Helsinki on “ethical principles for medical research involving human participants” (Preamble, Art. 1), such evaluation is required only when the medical research involves humanly identifiable material or data. In addition, the DigiCare4You project received the ethical approval number 34-19/11/2021 from the Harokopio University Research Ethics Committee.

## Results

### Overview

In this section, we present some preliminary descriptive evidence based on our dataset, (additional descriptive results are available in the Multimedia Appendix 4). The final dataset comprises responses from 260 HCWs situated in the 4 implementation countries of the DigiCare4You project; 102 respondents from Albania, 62 from Bulgaria, 68 from Greece, and 28 respondents from Spain. As more extensively discussed in the Multimedia Appendix 2, data notably exhibit some missing entries, as respondents had the option to skip questions they chose not to answer. Specifically, 3 respondents abstained from answering over half of the available questions; hence, we made the decision to exclude them from the analysis.

The distribution of sociodemographic characteristics across the 4 implementation countries is depicted in Table 3.

The reports include both the overall distribution of the variables and the stratified distribution by countries. Reported data include both absolute and relative frequencies, expressed in percentages. Upon examination of the table, it becomes evident that the majority of respondents were female (204/260, 78%), relatively young (171/260, 66% aged between 18 and 44 years old), and highly educated, with 200/260 respondents reporting having more than 16 years of education. Notably, 116/260 (45%) respondents were employed as medical specialists, followed by 62/260 (24%) respondents employed as general practitioners. Differences emerge when the 4 countries are compared. For instance, Albanian HCWs tend to be younger compared to their counterparts, while respondents from Spain exhibit the highest percentage of higher-educated respondents (26/28, 93% of the respondents declared having more than 15 years of education). Greece exhibits the highest percentage of respondents employed as dietitians or nutritionists (19/68, 28%) and health visitors (13/68, 18%)—qualified nurses who have additional training in public health and who offer support to families with young children. Albania has the highest percentage of respondents employed as general practitioners (40/102, 39%), followed by Spain and Bulgaria (7/28, 25% and 15/62, 24%, respectively).

**Table 3.** Sample sociodemographic characteristics overall and by country.

Characteristics	Albania	Bulgaria	Greece	Spain	All countries
Respondents, n (%)	102 (39)	62 (24)	68 (26)	28 (11)	260 (100)
Gender, n (%)					
Women	81 (79)	46 (74)	53 (78)	24 (86)	204 (78)
Men	20 (20)	15 (24)	15 (22)	4 (14)	54 (21)
Prefer not to answer	1 (1)	1 (2)	0 (0)	0 (0)	2 (1)
Age groups, n (%)					
18-29	52 (51)	15 (24)	23 (34)	4 (14)	94 (36)
30-44	30 (29)	10 (16)	26 (38)	11 (39)	77 (30)
45-59	19 (19)	30 (48)	15 (22)	11 (39)	75 (29)
60-75	1 (1)	7 (11)	4 (6)	2 (7)	14 (5)
Years of education, n (%)					
10-12	8 (8)	7 (11)	3 (4)	1 (4)	19 (7)
13-14	1 (1)	1 (2)	3 (4)	0 (0)	5 (2)
15-16	12 (12)	3 (5)	13 (19)	1 (4)	29 (11)
>16	81 (79)	46 (74)	47 (69)	26 (93)	200 (77)
Prefer not to answer	0 (0)	5 (8)	2 (2)	0 (0)	7 (3)
Employment, n (%)					
Dietitian or nutritionist	0 (0)	0 (0)	19 (28)	6 (21)	25 (10)
Health visitor	2 (2)	0 (0)	13 (19)	0 (0)	15 (6)
Medical specialist	52 (51)	23 (37)	30 (44)	11 (39)	116 (45)
General practitioner	40 (39)	15 (24)	0 (0)	7 (25)	62 (24)
Nurse	0 (0)	1 (2)	3 (4)	4 (14)	8 (3)
Other health allied professions	1 (1)	11 (18)	2 (3)	0 (0)	5 (14)
Prefer not to answer	7 (1)	12 (19)	1 (1)	0 (0)	20 (14)

### IRT Outcomes

As we have previously hinted throughout the paper, we used the information about the level of assessment of the 10 statements related to the HCWs’ attitude toward technology to predict their latent attitude in using such technologies. Specifically, we separately used the items related to the usage of digital technologies in working activities and to the usage of technological devices in the management of daily habits. The IRT models fit the data well, as all parameters resulted in being statistically significant, and all items contributed to explaining the latent traits. The complete estimation and fitting graphs are available in the Multimedia Appendix 5.

**Figure 1.**
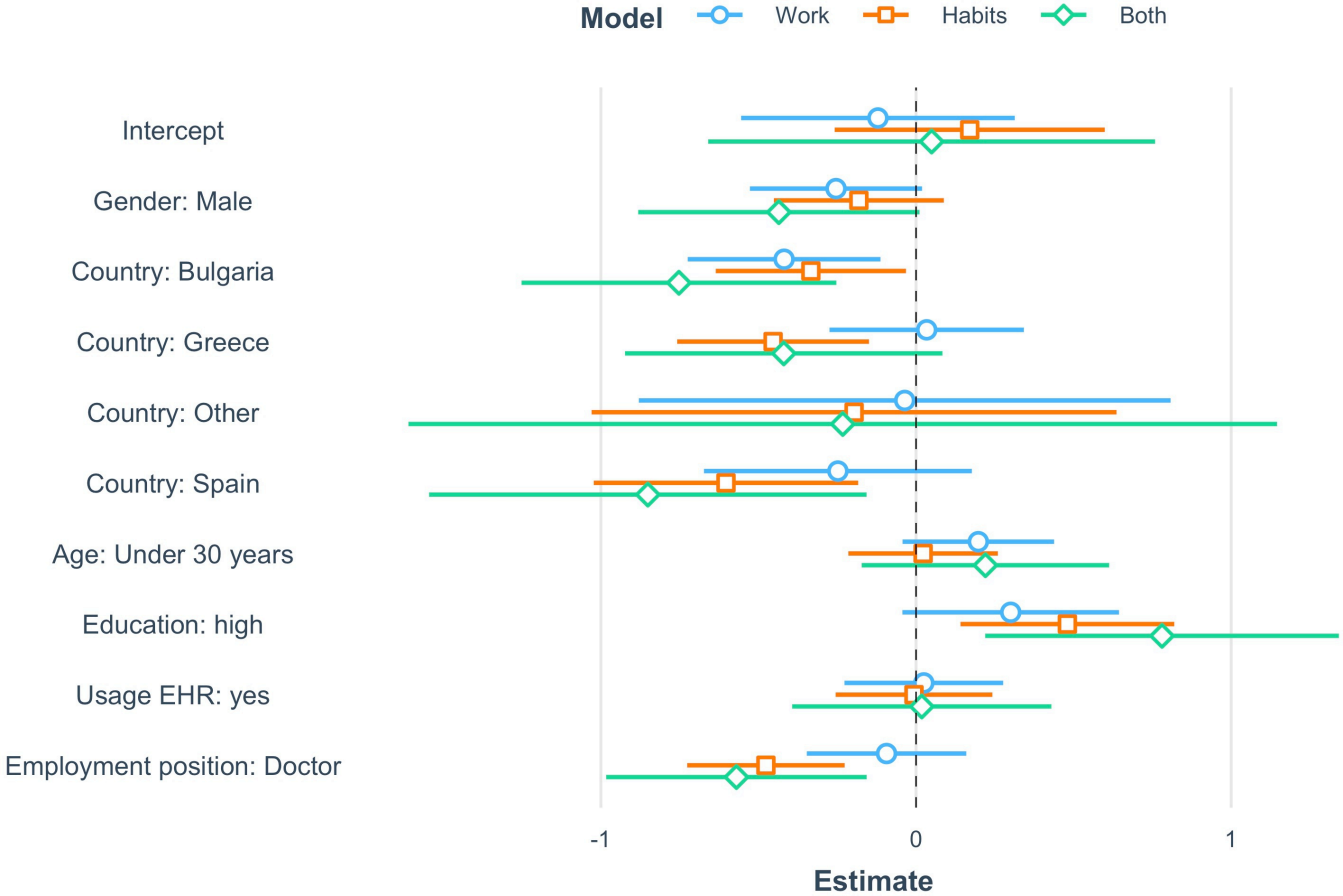
The determinants of the individual attitude toward using technology in the working activities, daily habits, and both. The figure reports estimates regarding the linear regression coefficient, together with their corresponding 95% CIs, in the 3 different models. EHR: electronic health record.

Once these traits were estimated, we explored whether socioeconomic factors have a role in determining their variability. Figure 1 graphically reports the main results of the 3 linear regression results in which the outcome variables were $\hat{\theta_{w}}$$\hat{\theta_{H}}$ and ${\hat{\theta }}_{WH}$ the latent attitudes of HCW toward using the technology during working activities, daily habits, and both, respectively. Complete tables are available in the Multimedia Appendix 5. In particular, Figure 1 provides an overview of the estimated coefficients in the 3 models, together with their corresponding 95% CIs. Results suggest that (1) males exhibit a less pronounced attitude in using technologies compared to women, and this effect is particularly significant when estimating the latent trait targeted at working activities ${\hat{\theta }}_{W}$ and the comprehensive latent trait ${\hat{\theta }}_{WH}$; (2) HCWs in Spain and Bulgaria demonstrate a lower propensity to use technologies compared to their Albanian counterparts; (3) being highly educated leads to a higher inclination to use technologies; and (4) doctors exhibit a lower propensity to use technologies compared to HCWs referred to other employment categories.

### DCE Outcomes

In this section, we reported the DCE results regarding the features that represent potential barriers or facilitators that may encourage or discourage HCWs in integrating a given digital health platform for patients’ data monitoring in their working routine. Moreover, we investigate whether their attitude toward the usage of technologies in working activities and in the management of their habits shapes their preferences about the ideal mHealth platform for patients’ data monitoring.

**Figure 2.**
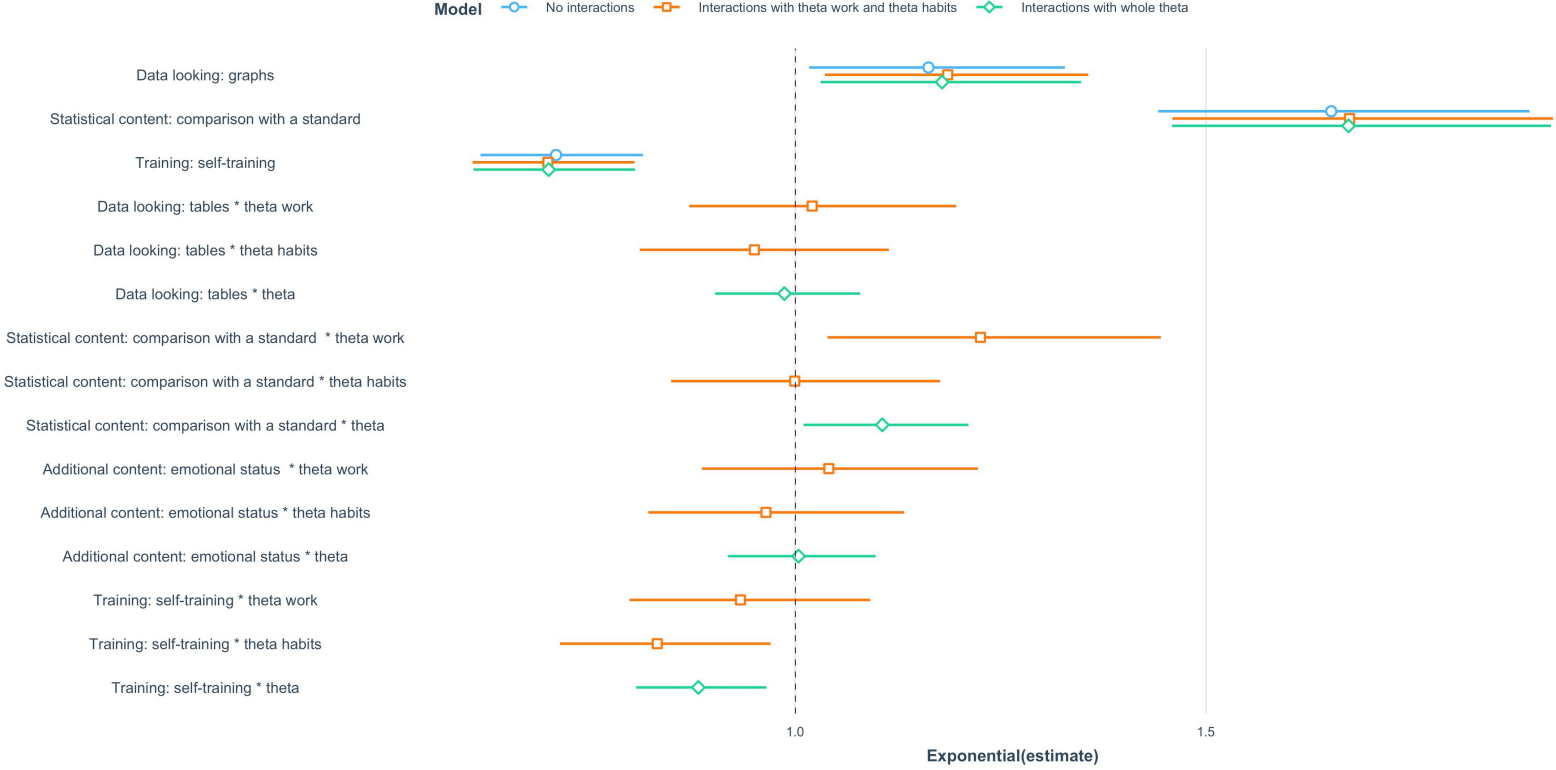
Discrete choice experiment results: odds ratios and corresponding 95% CIs of 3 conditional logit models.

In the DCE used in this setting, HCWs faced 6 pairwise comparisons (named Choice Sets), each presenting 2 alternative characterizations of the platform, which differed in the levels of their attributes. Figure 2 reports the main results of the DCE analysis, obtained by applying conditional logit models [57], where the response variable is the choice variable, and the regressors are the 4 attributes of the alternatives.

Specifically, to analyze data from the DCE, we run three conditional logit models. Figure 2 reports the estimated odds ratios, together with their corresponding 95% CIs of each model: (1) the first one (blue lines) provides the inclusion as independent variables of the 4 attributes only, (2) the second one (orange lines) also includes pairwise interactions between the 4 attributes and the 2 estimated latent traits ${\hat{\theta }}_{W}$ and ${\hat{\theta }}_{H}$, and (3) the third one (green lines) still includes interactions but with respect to the comprehensive latent trait ${\hat{\theta }}_{WH}$. Results here are presented in terms of odds ratios for ease of interpretation (odds ratios greater than 1 signal that the presence of a given feature encourages the respondents to choose that alternative). The coefficients related to the attributes that affect the HCWs’ choice of the ideal platform for patient data monitoring are highly significant.

By looking at the figure, we can definitely state that HCWs (1) prefer to analyze data through graphs rather than through tables, (2) find it very useful to have comparative statistics that relate data of their patients to a standard, rather than simply observing data of their patients, (3) prefer to have information on the patients’ clinical history rather than on their emotional status, and (4) prefer to receive an active group training on the platform rather than completing self-tutorials.

By looking at the interactions, we can also state that (1) having a higher attitude in using technologies in working activities (${\hat{\theta }}_{W}$) and having a high comprehensive attitude toward technology (${\hat{\theta }}_{WH}$) significantly increases the preference of having statistical data about the comparison of patients against a standard, instead of having data related to the given patient only; and (2) an increased attitude toward the usage of technological devices in managing lifestyle habits (${\hat{\theta }}_{H}$) and toward technologies as a whole (${\hat{\theta }}_{WH}$) decreases the preference of having a self-training program to learn the platform, instead of a group training.

**Figure 3.**
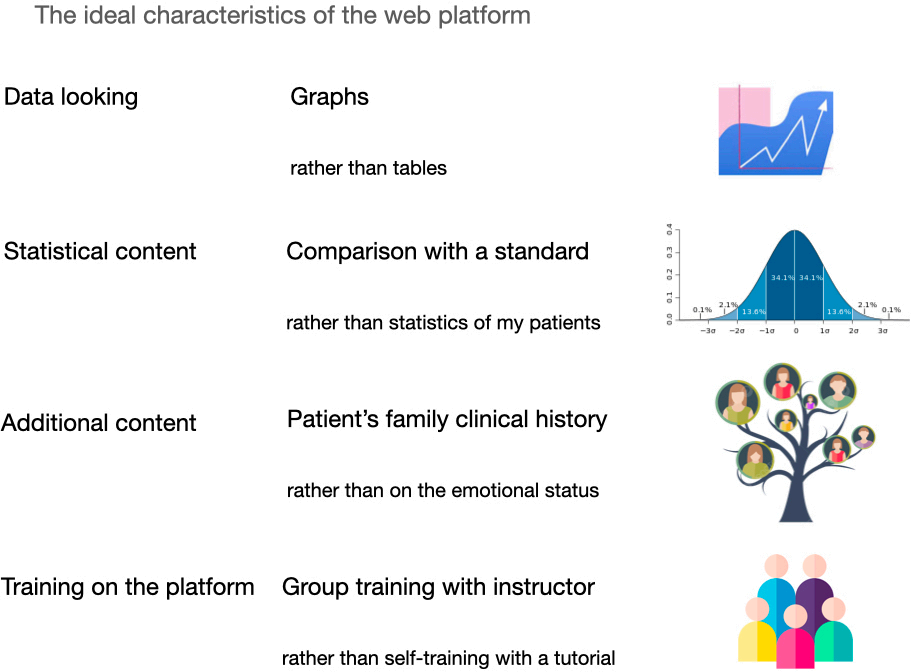
Discrete choice experiment results: ideal characteristics of the web platform.

As a consequence, the ideal digital health platform for patient data monitoring should provide intuitive graphs, statistics that compare patients’ data against a standard, information about the patients’ family clinical history, and health workers should be trained for the usage of that platform through a group training led by an instructor. The characteristics of the ideal platform chosen by HCW for patient data monitoring are represented in Figure 3.

## Discussion

### Principal Findings

This contribution proposes an integrated approach based on IRT and DCE (1) to evaluate the HCWs’ attitude toward the usage of technological devices in their working activities and in managing their lifestyle habits, (2) to explore the role of sociodemographic characteristics in shaping these attitudes, and (3) to appraise whether their attitude toward technology shapes their preferences about a novel digital health platform for patient data monitoring. First, results suggest that health workers show an overall positive attitude toward the usage of technological devices both in their working activities and in their lifestyle habits. This propensity is particularly strong for women and highly educated professionals, while it appears to be weaker for doctors compared to other health care professionals. Similarly to the study by Muiruri et al [17], the employment position plays a role in determining the attitude toward technology.

The different country of the respondents also affects the attitude toward technology, as Albanian HCWs have a stronger propensity to integrate technological tools within their daily routines compared to the other countries. These findings are in line with the review done by Borges et al [26], who identified that country-specific technologies can affect the adoption of digital health devices. Thus, statistically different results may be hypothesized as the respondents being a proxy of their countries’ readiness to adopt these technologies.

In some studies [17], sociodemographic variables, such as gender and education, do not play a role in determining the HCWs’ willingness to adopt technologies, while in our contribution, they result in significantly impacting the HCWs’ attitude toward technology, as already pointed out in Borges do Nascimento et al [26]. Second, the DCE results suggest that the type of training, the way data are presented, and the information provided by the platform play a pivotal role in shaping the HCWs’ willingness to adopt a digital platform for patients’ data monitoring. The inclination for personal training instead of self-guided tutorials was already pointed out before [4,19,20], besides the findings of [21,26], indicating a concern with the quality of the training received by the staff.

The preferences regarding data looking in the format of graphs and statistics compared to a standard hint to preferences aligned with more intuitive and user-friendly interfaces, which are also previously mentioned in other studies [4,14,20,21] as facilitators of the adoption of digital health tools by the HCW.

To the best of our knowledge, there are no existing studies that jointly explore the HCWs’ attitude toward technology and their preferences about such digital technologies. Our findings suggest that there are relevant interactions between these 2 aforementioned topics, and this issue should be further explored, as it provides a global understanding of the approach of HCWs toward digital health systems.

### Strengths and Limitations

This work has some limitations. The main limitation is that the sample of individuals asked to complete the WCS might be affected by a selection bias due to the snowball sampling nonprobabilistic design. The sampling technique was chosen based on the availability of resources in the 4 countries under study. An additional limitation of the study is that the sample size in each of the 4 countries, even if it is relatively large, does not allow us to conduct a stratified analysis, where we separately implement the integrated analytical framework in each country and then we inspect differences among countries. The relatively low response rates and participation, especially in some countries like Spain, can be due to people’s fatigue with invitations and participation in various surveys (oversurveying), especially after the COVID-19 period of dominance of e-communication. However, this work also offers valuable contributions. First, it proposes an integrated analytical approach based on IRT and DCE to simultaneously investigate the HCWs’ attitude toward the usage of technologies, the HCWs’ preferences about potential digital tools to be integrated within their working routine, and potential interactions between the aforementioned topics. This approach empowers us to delve into the intricate dynamics between HCWs’ fundamental attitudes toward technology and their specific inclinations toward the novel platform. This exploration adds a layer of depth to our understanding, allowing for a more comprehensive assessment of the factors influencing their preferences. Second, this study covers a wide international cohort of HCWs enrolled in multiple European countries. Therefore, it does not focus on specific experiences that refer to empirical contexts that are likely to be narrow both from the spatial and the temporal perspective but attempts to obtain findings that can be potentially generalized.

### Conclusions

The analytical framework presented in this work, merging IRT and DCE, elevates our comprehension of HCWs’ propensities regarding the integration of digital health tools. The breadth of the analysis, encompassing a wide international cohort of HCWs, places the results as not just informative but also highly applicable across a spectrum of contexts. This potential generalizability underscores the significance of the findings, making them a relevant resource for shaping strategies and targeting interventions in various health care settings.

The findings from this study have significant policy implications for the integration of digital systems in health care systems. First, the study reveals that HCWs generally hold a positive attitude toward the use of technological devices, emphasizing the potential benefits of these tools in enhancing health care efficiency. It follows that political authorities should push for the progressive integration of digital health systems, integrating patient-generated data into the daily workflows of health care workers to enhance care delivery by improving the available infrastructures through which digital tools are implemented and promoting laws and programs to encourage the gradual development of a technologically empowered health care system. Second, the research highlights the influence of socioeconomic factors in shaping health workers’ attitudes toward digital health tools. Policymakers should take into account these factors when designing strategies for the adoption of such technologies, targeting specific support to those categories that have a weaker propensity for adopting these technologies. Furthermore, the study underscores the importance of considering specific characteristics of platforms for patients’ data monitoring, as these features significantly impact HCWs’ willingness to integrate them into their working routines. Despite the relevance of our results, the attitude of HCWs toward digital tools and their preferences toward the ideal characteristics of these tools should be further explored in future studies to enhance the global understanding of this topic and to provide even stronger evidence for policymakers. Future works may consider wide international samples. In addition, researchers should deeply understand which are the organizational and infrastructural barriers that still limit the development of digital health systems and which are the possible approaches to enhance the HCWs’ attitude toward these technologies.

## Supplementary material

10.2196/67142 Multimedia Appendix 1 The Web Conjoint Survey.

10.2196/67142 Multimedia Appendix 2 Analysis of missing data.

10.2196/67142 Multimedia Appendix 3 Focus groups.

10.2196/67142 Multimedia Appendix 4 Additional descriptives.

10.2196/67142 Multimedia Appendix 5 Item response theory.

10.2196/67142 Checklist 1 CHERRIES checklist.

## Abbreviations

DCE: discrete choice experiment
GRM: Graded Response Model
HCW: health care worker
IRT: item response theory
PMM: Predictive Mean Matching
WCS: Web Conjoint Survey
