# Supplementary material for: Understanding Health Care Workers’ Attitudes and Preferences Toward Digital Patient Monitoring Platforms: Cross-Country Survey Study
Source: JMIR Form Res. 2025 Sep 23;9:e67142. doi: 10.2196/67142 (PMC12456460; doi:10.2196/67142)
Supplement: Multimedia Appendix 1 [file formative-v9-e67142-s001.docx]

# The Web Conjoint Survey

The Web Conjoint Survey (WCS) proposed to the respondents is composed by the following questions. Please cite this work and refer to the DigiCare4You project if you use questions included in this survey for research purposes.

## B.1 Basic Demographics

1. What gender do you identify as?
   - Male
   - Female
   - Non-binary - Prefer not to answer
2. What is your age?
   - 18 - 29 years old
   - 30 - 44 years old
   - 45 - 64 years old
   - 65 - 74 years old
   - Prefer not to answer
3. Please declare the years of education you have successfully completed
   - 10-12 years
   - 13-14 years
   - 15-16 years
   - >16 years
   - Prefer not to answer
4. What is your current employment position?
   - Nurse
   - Physician/ General Practitioner
   - Medical Specialist (Specialization:)
   - Health visitor
   - Dietitian/ Nutritionist
   - Social worker
   - Healthcare Assistant
   - Other: please define

## B.2 Baseline attitude towards technology

- Do you use Electronic Health Record (EHR) system(s) to enter or review patient information

(AHRQ survey)

- - Yes
  - No
  - Not applicable (I do not have any device to keep electronic records)

- Prefer not to say

- (Use of technological tools in working life) On a scale from 1 to 10, rate your level of agreement with the following statements:
  1. I think that using health technologies (e.g electronic health records, mhealth, telehealth) in my job would enable me to accomplish tasks more quickly
  2. I think that using health technologies (e.g electronic health records, mhealth, telehealth) would make it easier to do my job
  3. I would find health technologies (e.g electronic health records, mhealth, telehealth) useful in my job
  4. I think that using health technologies (e.g electronic health records, mhealth, telehealth) would improve the quality of the work I do
  5. I think that using health technologies (e.g electronic health records, mhealth, telehealth) in my job would increase my productivity
- (Use of technological tools in managing lifestyle) On a scale from 1 to 10, rate your level of agreement with the following statements:
  1. I use to / I would like to track some health parameters (e.g hearth rate, Oxygen saturation, quality of sleep,) through a technological device
  2. I like to track my lifestyle habits through a mobile app
  3. I like to monitor what I eat and drink through a mobile app
  4. I like to track the statistics regarding some activity parameters (steps taken, stairs climbed, minutes of workout performed) through a technological device.
  5. I like to write on a virtual notebook some notes about what I do in a day and how I feel

## B.3 Discrete Choice Experiment

*Imagine that in your working activity you are offered to use a novel digital platform, reporting on the lifestyle habits of your chronic patients and you can choose between two types of platforms, which one would you choose?*

- DATA LOOKING The way the health worker prefers to look at the data a) Tables b) Graphs
- STATISTICAL CONTENT Statistical content the user would like to see in the platform a) Statistics of my patients b) Statistics of my patients against a standard (national average, app users average)
- ADDITIONAL CONTENTS Which additional content the health worker wishes to analyze on the platform a) Patients family clinical history b) Patients emotional status.
- TRAINING ON THE USE OF THE PLATFORM Type of training on the use of the platform for health workers a) Group training with instructor b) Self-training with tutorial
