# Supplementary material for: Understanding Health Care Workers’ Attitudes and Preferences Toward Digital Patient Monitoring Platforms: Cross-Country Survey Study
Source: JMIR Form Res. 2025 Sep 23;9:e67142. doi: 10.2196/67142 (PMC12456460; doi:10.2196/67142)
Supplement: Multimedia Appendix 2 [file formative-v9-e67142-s002.docx]

## Multimedia Appendix 2: Analysis of missing data

This Section provides some details about the presence of missing entries in the data. Figure 1 illustrates the complete data structure, with each cell in the plot corresponding to a cell in the dataset. Numeric variables are depicted in light blue, while factors are represented in red; in both cases, missing entries are denoted in grey. Note that the variables denoted as Work [number] pertain to statements measuring attitudes toward using technological devices in professional life. Similarly, variables labeled as Habits [number] are associated with statements gauging attitudes toward using technological devices in managing lifestyle habits. The notation DCE [number] is employed for variables linked to responses in the Discrete Choice Experiments (DCE) comparisons. Notably, it can be observed that missing entries predominantly impact questions related to the baseline attitude toward technology.


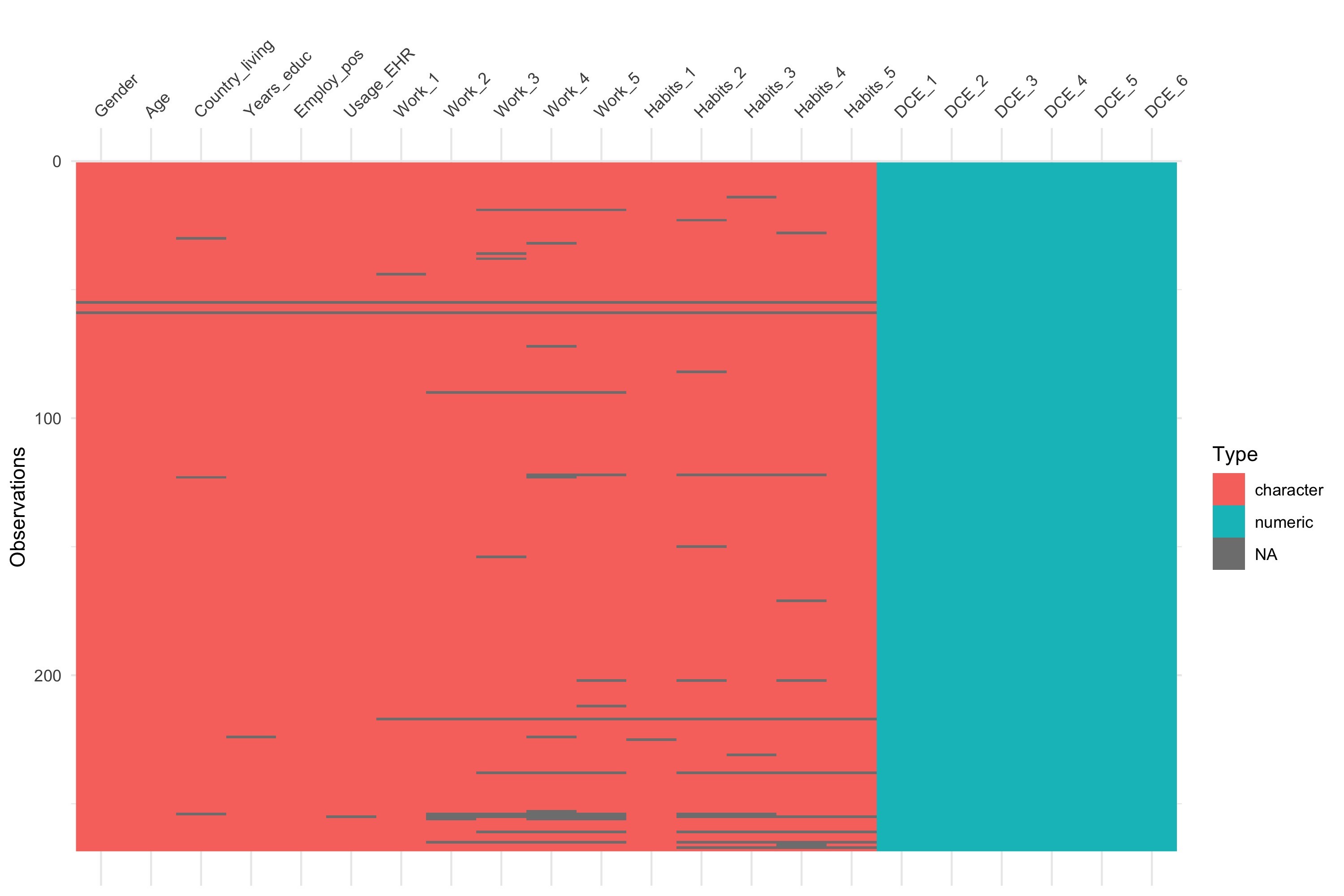


Figure 1: Missing data: overview

Figure 2 explores the missingness by two slightly different perspectives: the left side Figure (2a) provides information about the number of missing entries in each questionnaire, while the right side Figure (2b) plots the percentage of missing entries observed in each variable (question).


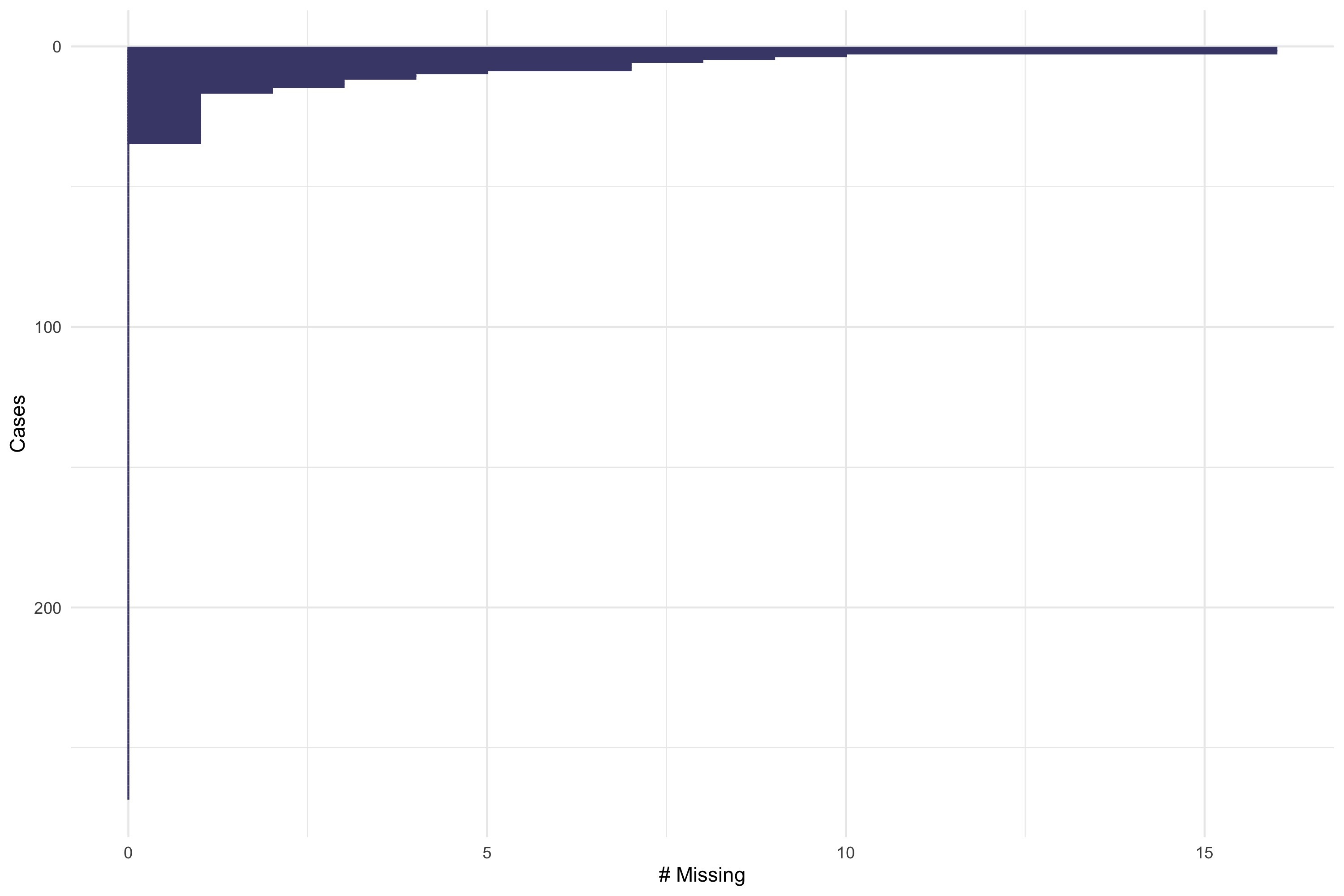

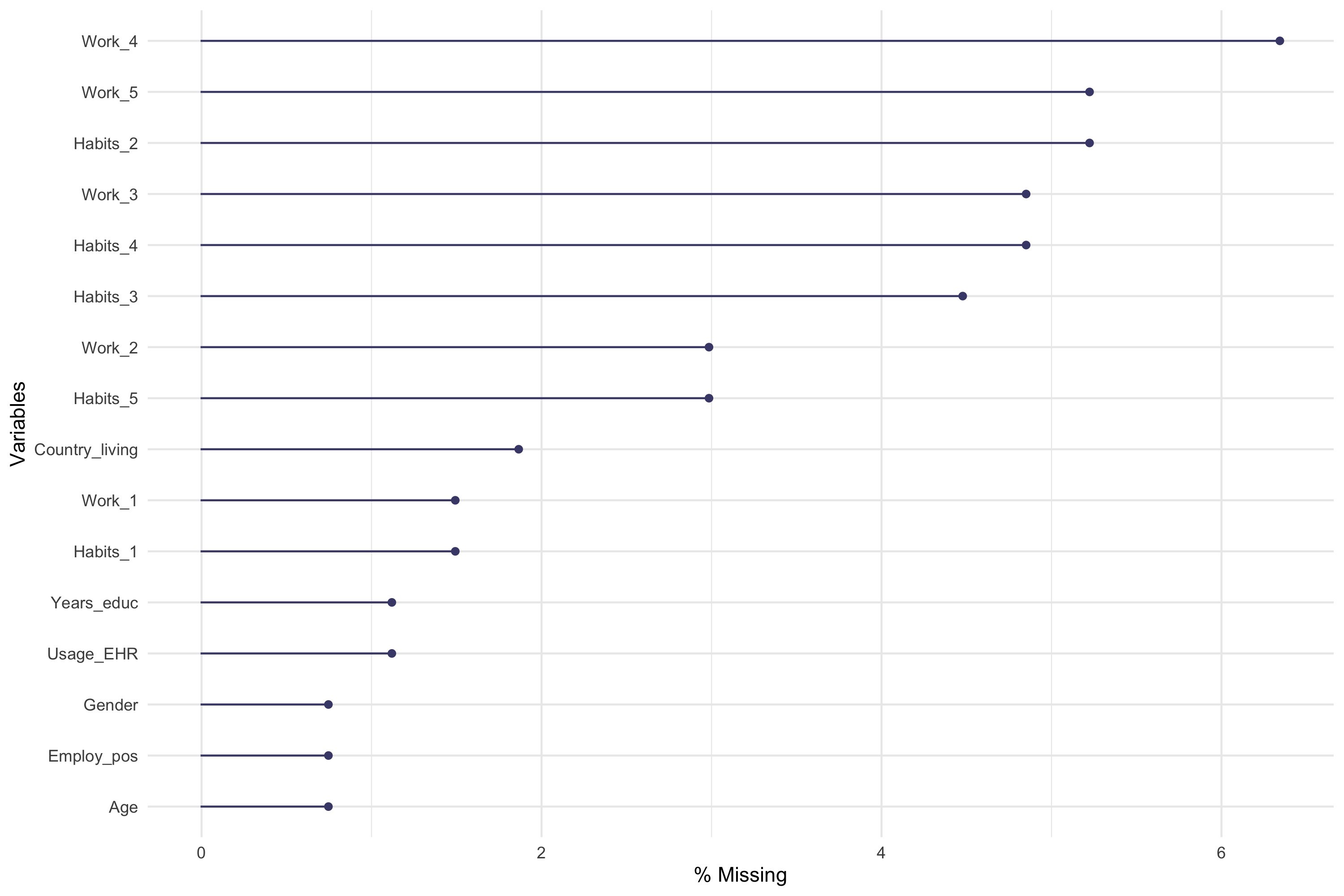


(a) Individuals (b) Variables

Figure 2: Missing data: variables’ vs individuals’ perspective

Finally, Figure 3 provides an intuition about the most prevalent patterns of jointly missing variables.


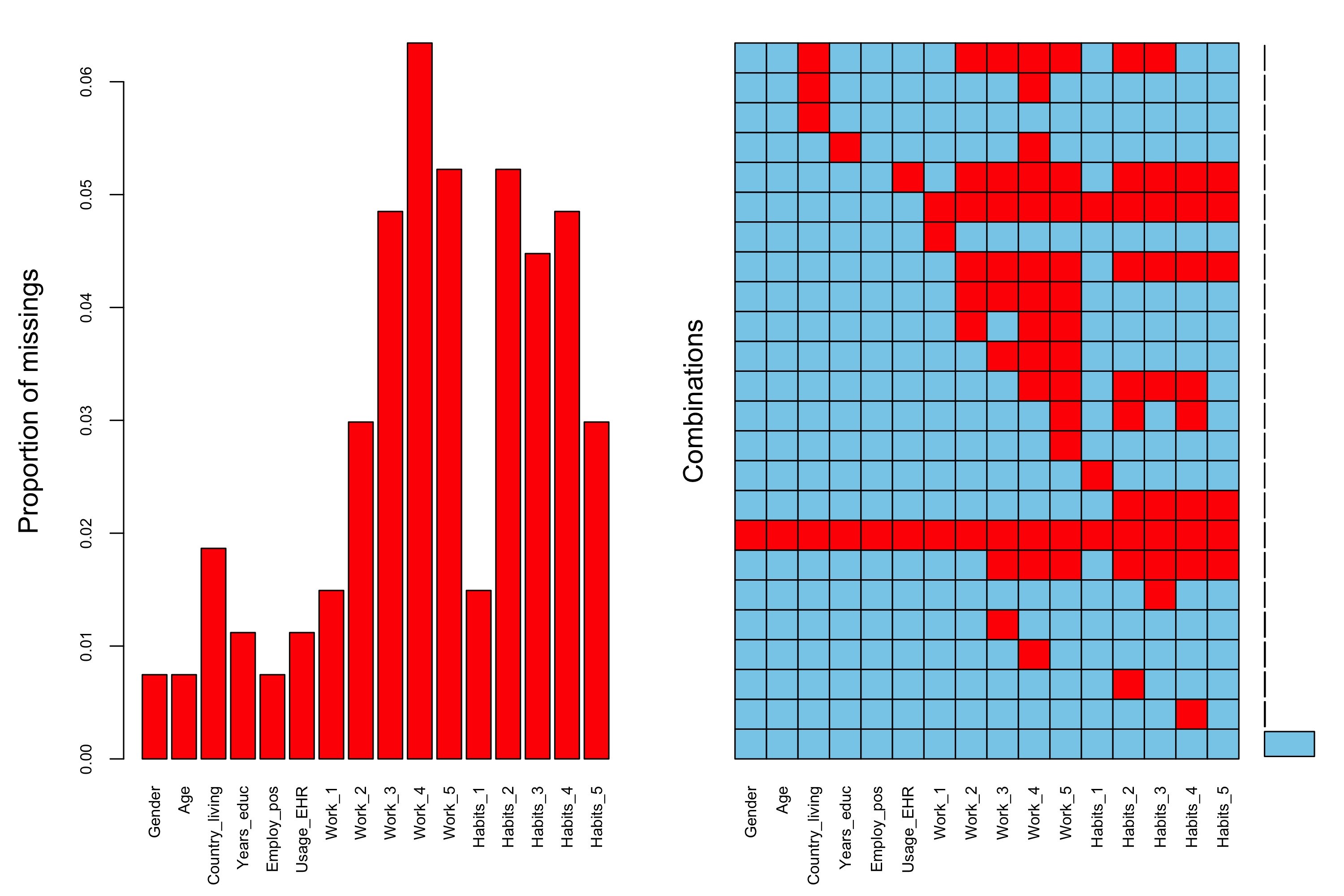


Figure 3: Proportions and combinations of missing data. The left side figure shows the percentage of missing entries in the variables; the right side figure describes the most prevalent patterns of variables that are likely to be jointly missing.

Additionally, Table 1 reports the number of missing entries in the variables related to the assessments for measuring the HWs’ attitude towards technology. As it is possible to observe, the missing entries are relatively few, and mainly impact the statements about the incorporation of technologies in working life.

Table 1: Individuals attitude towards technology: missing entries

|  | Mean | Standard Deviation | # Missings |
| --- | --- | --- | --- |
| Work 1 | 8.41 | 2.23 | 1 |
| Work 2 | 8.68 | 1.90 | 5 |
| Work 3 | 8.63 | 2.05 | 10 |
| Work 4 | 8.51 | 1.93 | 14 |
| Work 5 | 8.35 | 2.12 | 11 |
| Habits 1 | 8.16 | 2.36 | 1 |
| Habits 2 | 6.98 | 2.95 | 11 |
| Habits 3 | 5.59 | 3.17 | 9 |
| Habits 4 | 7.54 | 2.78 | 10 |
| Habits 5 | 5.74 | 3.24 | 5 |

As described in the core of the paper, we impute missing entries Here, we compare the distributions of original and imputed data using some useful plots. In particular, Figure 4 enables us to compare the distributions of original and imputed data according to two slightly different perspectives: the left side figure produces a scatter plot related to the distribution of the variables i) in original data with no imputation (0) and ii) in the completed dataset once that imputation is done (1); the right side figure shows instead a comparison between densities, where the density of the imputed data is showed in magenta while the density of the observed data is showed in blue. Ideally, we should observe a correspondence in the shape between the magenta points (representing imputed values) and the blue points (representing observed values). A congruent shape between the two sets indicates that the imputed values are genuinely *plausible*. The difference in the distributions observed in the right side figure is due to the very small number of missings in the original data, but the left side figure comparing the overall distributions suggests that imputed data are plausible.


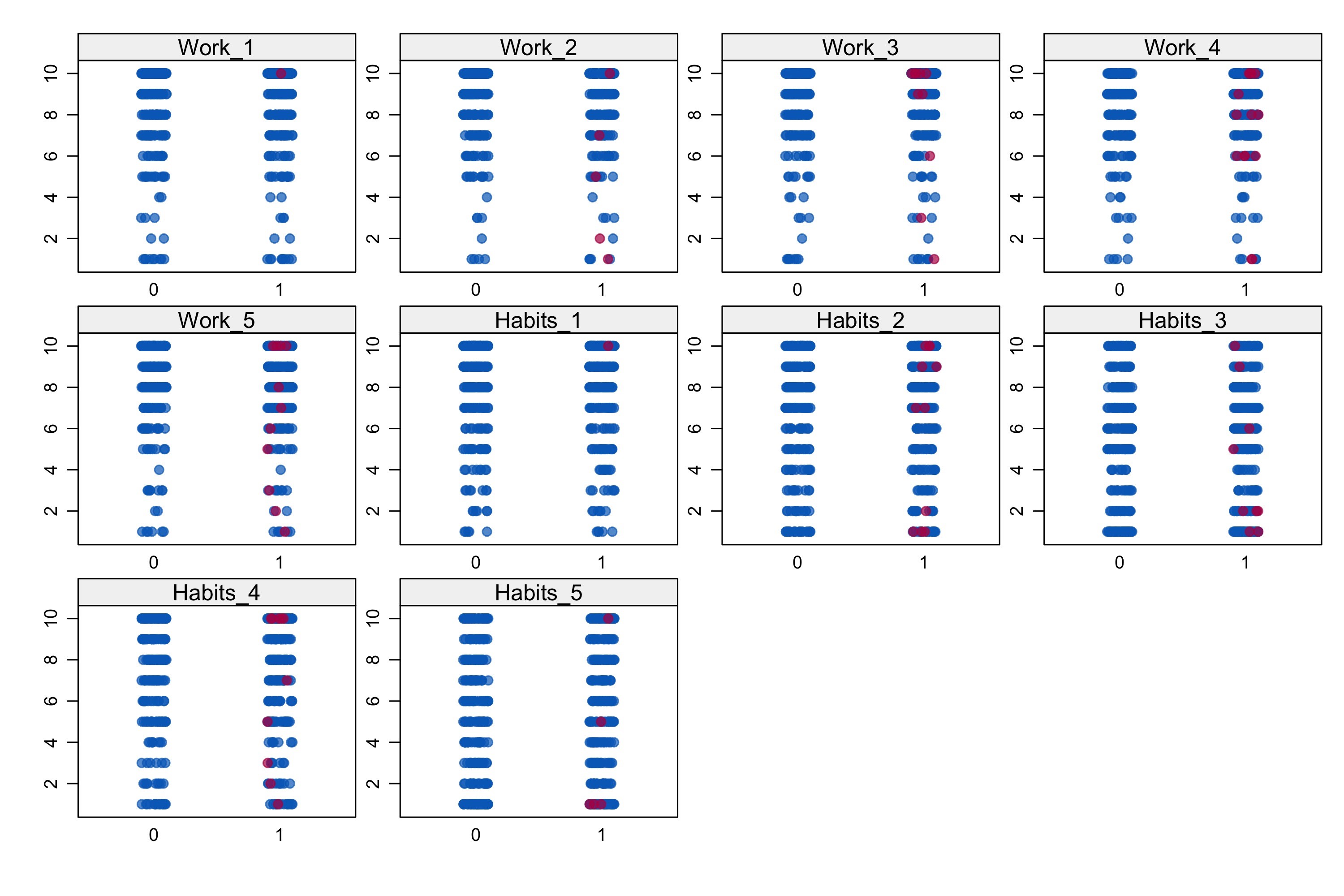

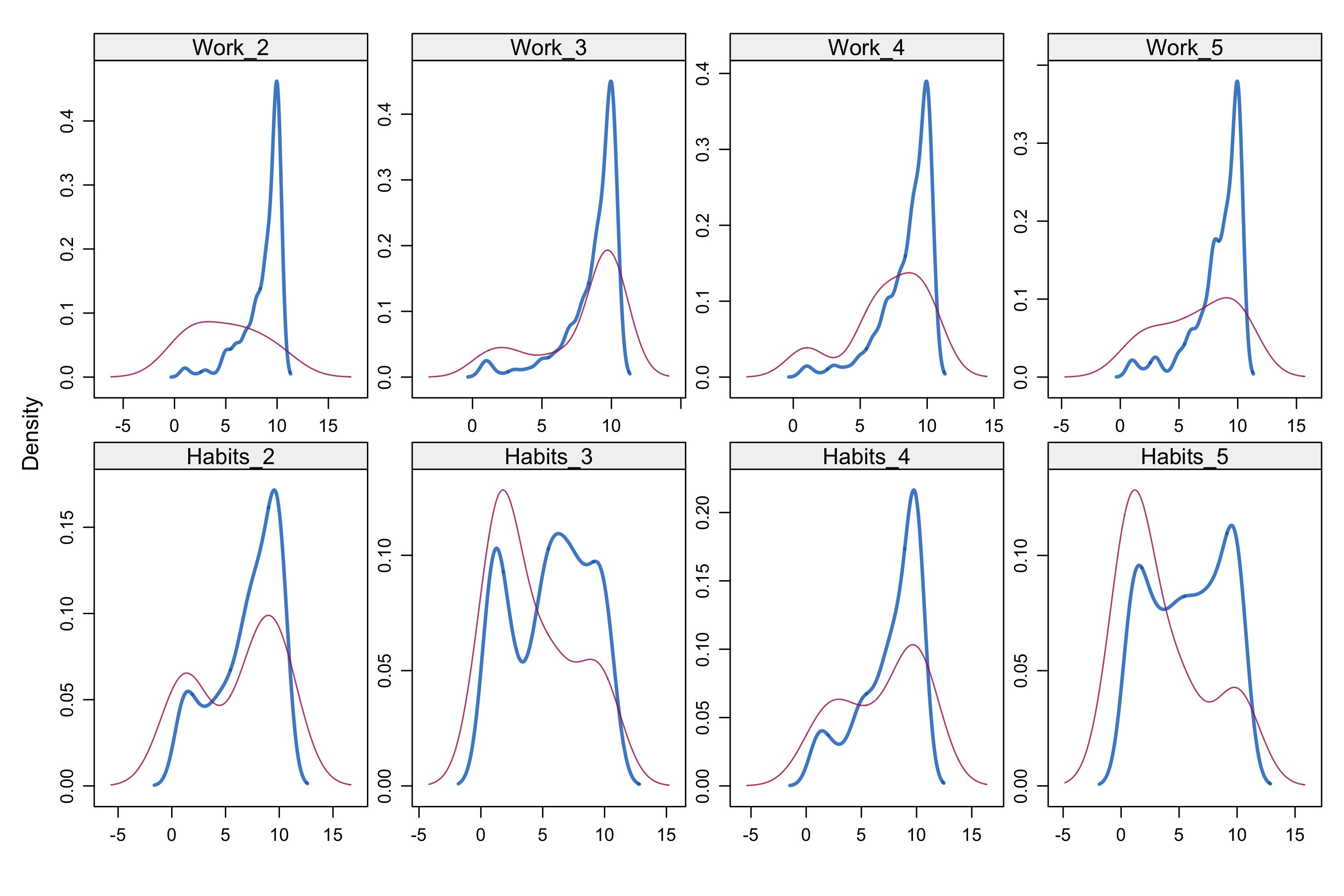


(a) Working activities (b) Distribution

Figure 4: Observed vs imputed data
