# Supplementary material for: Understanding Health Care Workers’ Attitudes and Preferences Toward Digital Patient Monitoring Platforms: Cross-Country Survey Study
Source: JMIR Form Res. 2025 Sep 23;9:e67142. doi: 10.2196/67142 (PMC12456460; doi:10.2196/67142)
Supplement: Multimedia Appendix 3 [file formative-v9-e67142-s003.docx]

## Multimedia Appendix 3: The focus groups

### Idea

This section provides an overview of the setup and organization of focus groups within the Digicare4You project, offering a comprehensive analysis of the primary outcomes. A focus group is a moderated discussion among a small group of individuals focused on a specific issue (Morgan; 1996; Smithson; 2008; Liamputtong; 2011). The participants are carefully selected based on pre-defined criteria to ensure a representative sample of the target population (Parker and Tritter; 2006). Notably, the role of the moderator is typically assigned to an external professional. Employed widely across various disciplines such as marketing, psychology, political science, and sociology, focus groups serve as a qualitative research methodology (Thomas et al.; 2000; Cyr; 2019). In contrast to solely examine participants’ responses to specific questions, researchers utilizing focus groups to fully delve into participants’ behaviors, body language, and the intricate social dynamics that emerge during group discussions. The significance of focus groups in social sciences lies exactly in their ability to reveal the nuanced social dynamics arising from participant interactions, a facet which is often challenging to capture through individual interviews. This methodology offers a unique perspective, allowing researchers to investigate not only what participants express verbally but also the meaning of their non-verbal signals and the collective dynamics shaping the meeting. Focus groups were organized within the DigiCare4You Project to understand the potential barriers and facilitators for HWs in incorporating the mHealth digital tool into their work activities, specifically for monitoring data related to their patients.

### Set Up

In the DigiCare4You Project, focus groups were organized by leading partners in the four implementation countries. Conducted between November and December 2021, each session lasted 60-90 minutes and was held in the local language to facilitate participants’ expression of opinions. The local organizers, responsible for participant selection, chose approximately 10-12 policymakers and health workers for each group. An external moderator and co-moderator, designated by the organizers, guided discussions, ensuring clarity and encouraging participants to elaborate on their opinions. Before initiating discussions, participants signed a Consent Form. Discussions were audio-recorded to facilitate accurate transcription, and participants had the freedom to skip questions or withdraw from the discussion at any time. Following the collection of basic demographic information, including age, gender, and employment role, moderators posed specific questions to participants. Health workers (HWs) and healthcare policy makers (HPMs) shared their opinions on the availability and quality of human resources and infrastructure for DigiCare4You implementation. Additionally, discussions captured implementers’ perspectives on potential barriers and facilitators for integrating the DigiCare4You mhealth tool for patients data monitoring into their working practices practices in a cost-effective and sustainable manner.

### Results

The focus groups were well-attended, and all received positive feedback from the participants. HWs enthusiastically embraced the DigiCare4You Project, expressing their support and satisfaction with the initiative. The discussion provided relevant insights into the preferences of HWs regarding the characteristics of the DigiCare4You solution and contributed to understand the existing human resources and infrastructures that may be helpful for the implementation of the DigiCare4You solution.

Table 1 provides information on the participants in the focus groups for each implementation country. As observed from the table, all focus groups have a sufficient number of participants employed as HWs or HPMs.

Table 1: Focus groups: participants

| Country | Participants | Gender | Role |
| --- | --- | --- | --- |
| Albania | 23 | 19 Women, 4 men | 6 HPMs; 17 HWs |
| Bulgaria | 9 | 7 Women, 2 men | 5 HPMs; 2 HWs; |
| Greece | 12 | 8 Women, 4 men | 8 HPMs; 4 HWs |
| Spain | 13 | 13 Womwn | 10 HPMs; 2 HWs |

Participants in the focus group unanimously expressed the belief that the integration of novel technologies, specifically in the form of web platforms for patients data monitoring, is extremely useful. They stated that these technologies could not only help them monitor their patients but also assist patients themselves in adopting and maintaining a healthy lifestyle. They emphasized its potential in preventing or delaying chronic diseases. The perceived effectiveness of such technology is heightened when it offers comprehensive lifestyle information of patients, such as assessments of physical activity and sleep quality. HWs asserted that a similar technology, whether in the form of a computer platform, could greatly assist them in remotely monitoring their9 patients. This functionality would streamline their daily routines, allowing for more efficient handling of diverse data sources, for constant patient connectivity, for a reduction in in-person visits, and for a increased individualized attention to each patient. Emphasizing the importance of user-friendliness, language accessibility, and interactivity, HWs expressed the importance for a platform of presenting comprehensive and intuitive data. They stressed the importance of avoiding excessive notifications while retaining direct communication channels between health workers and patients.

## References:

Cyr, J. (2019). *Focus groups for the social science researcher*, Cambridge University Press.

Liamputtong, P. (2011). Focus group methodology: Principle and practice, *Focus Group Methodology* pp. 1–224

Morgan, D. L. (1996). Focus groups, *Annual review of sociology* **22**(1): 129–152.

Parker, A. and Tritter, J. (2006). Focus group method and methodology: current practice and recent debate, *International Journal of Research & Method in Education* **29**(1): 23–37.

Smithson, J. (2008). Focus groups, *The Sage handbook of social research methods* pp. 357–370.

Thomas, M., Frankland, J. and Bloor, M. (2000). Focus groups in social research, *Focus Groups in Social Research* pp. 1–120.
