# Supplementary material for: Understanding Health Care Workers’ Attitudes and Preferences Toward Digital Patient Monitoring Platforms: Cross-Country Survey Study
Source: JMIR Form Res. 2025 Sep 23;9:e67142. doi: 10.2196/67142 (PMC12456460; doi:10.2196/67142)
Supplement: Multimedia Appendix 4 [file formative-v9-e67142-s004.docx]

## Additional descriptives

The second section of the survey delves into the inclination of HWs towards the utilization of technological devices. Specifically, respondents are asked to express their level of agreement with ten statements regarding the incorporation of digital tools into their professional activities and the management of their lifestyle habits. Additionally, participants are encouraged to share their experiences with Electronic Health Records (EHR), detailing whether they have any experience with the usage these electronic system for entering or reviewing patient data. Figure 1 summarizes the main results related to these questions. In particular, it reports the distribution of the usage of EHR over countries and years of education (left side figure), and the pirate plot representing the distribution of the overall score obtained in the items over countries -the total score is simply given by the sum of the levels of assessments in the ten items - (right side figure). Pirate plots inspect both the densities and the quantiles of the distributions and results suggest that Spanish HWs are those who are more likely to have previous experience with EHR but also those who have the lowest average score measuring their overall attitude in using technology in working activities and in the management of their lifestyle habits. Conversely, health workers living in Albania tend not to have previous experience with EHR, but show a positive attitude towards the usage of technological devices.


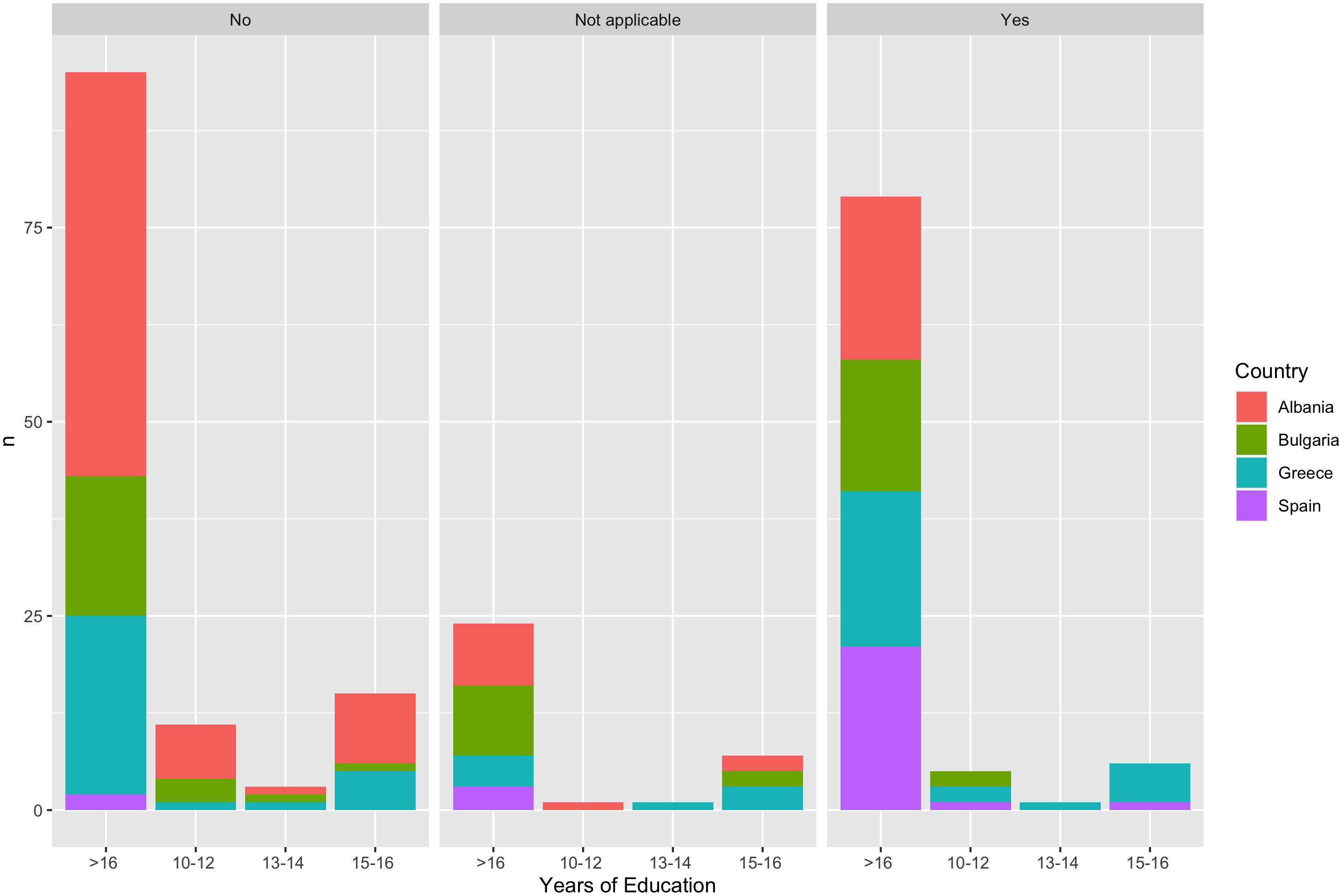

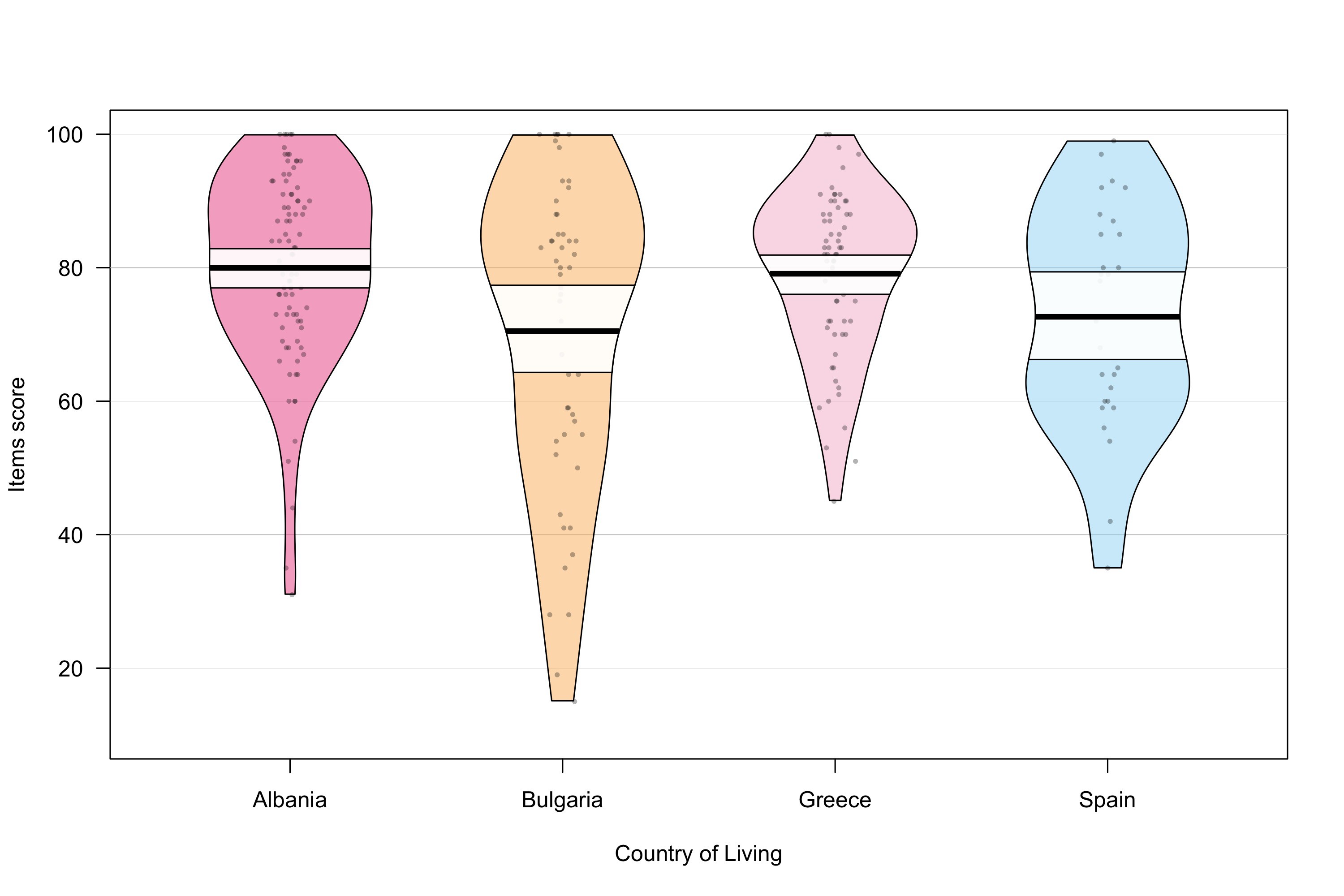


(a) Previous experience with EHR (b) Pirate plot

Figure 1: Overall attitude towards technology
