# Supplementary material for: Understanding Health Care Workers’ Attitudes and Preferences Toward Digital Patient Monitoring Platforms: Cross-Country Survey Study
Source: JMIR Form Res. 2025 Sep 23;9:e67142. doi: 10.2196/67142 (PMC12456460; doi:10.2196/67142)
Supplement: Multimedia Appendix 5 [file formative-v9-e67142-s005.docx]

# Individual attitude towards technology: additional evidence

## The items

Table 1 collects graphs representing pirate plots related to the distribution of the items related to the HWs’ attitude towards technology, across countries. It is possible to observe that Bulgarian respondents present a more skeptical attitude towards technology, especially for assessments related to the incorporation of technologies in working activities. Albanian HWs tend to be more inclined to incorporate technologies in both their working life and their private life, but still there are some negative outliers whose baseline attitude towards technology is substantially lower.

Table 1: Individuals attitude towards technology: pirate plots

| 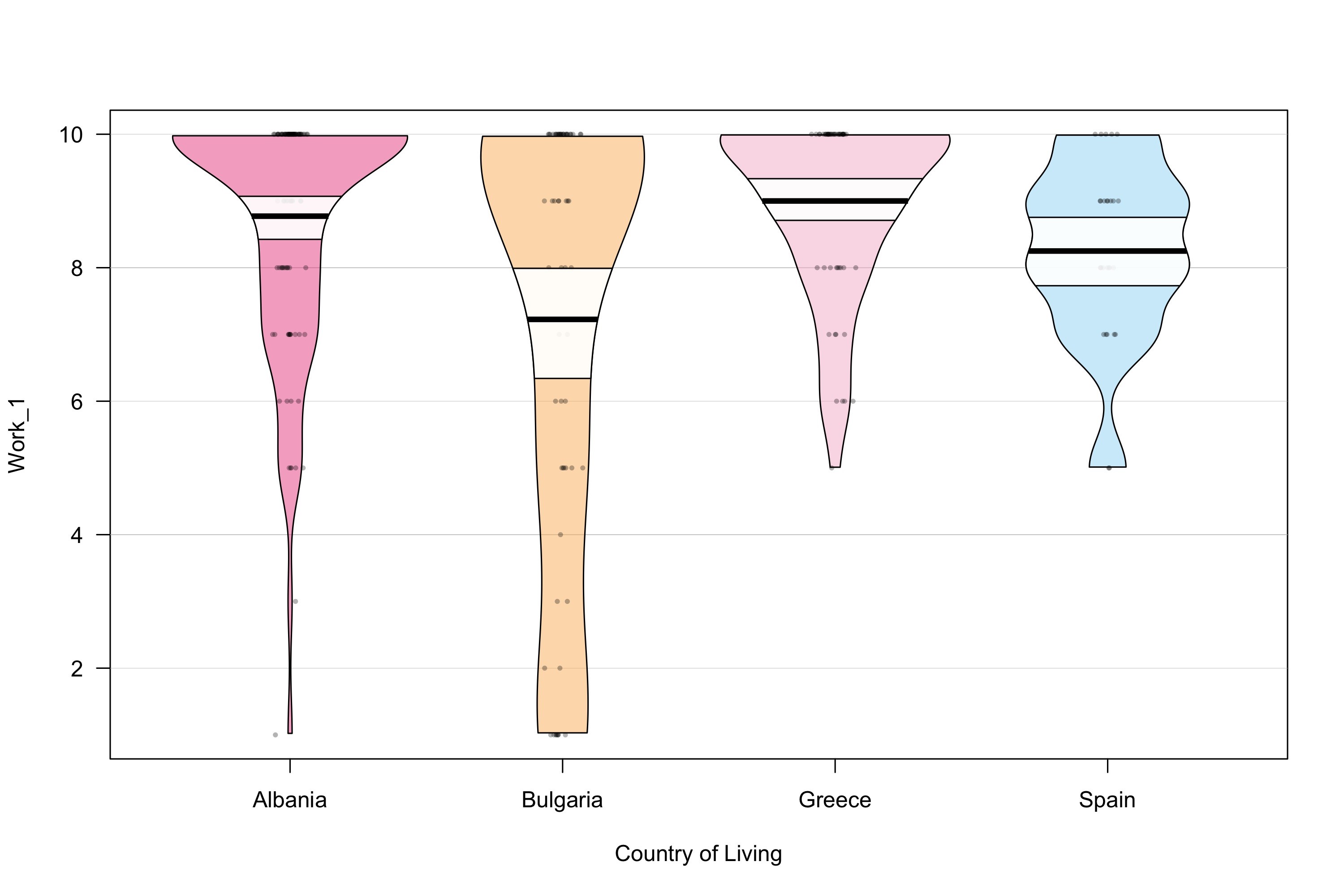 | 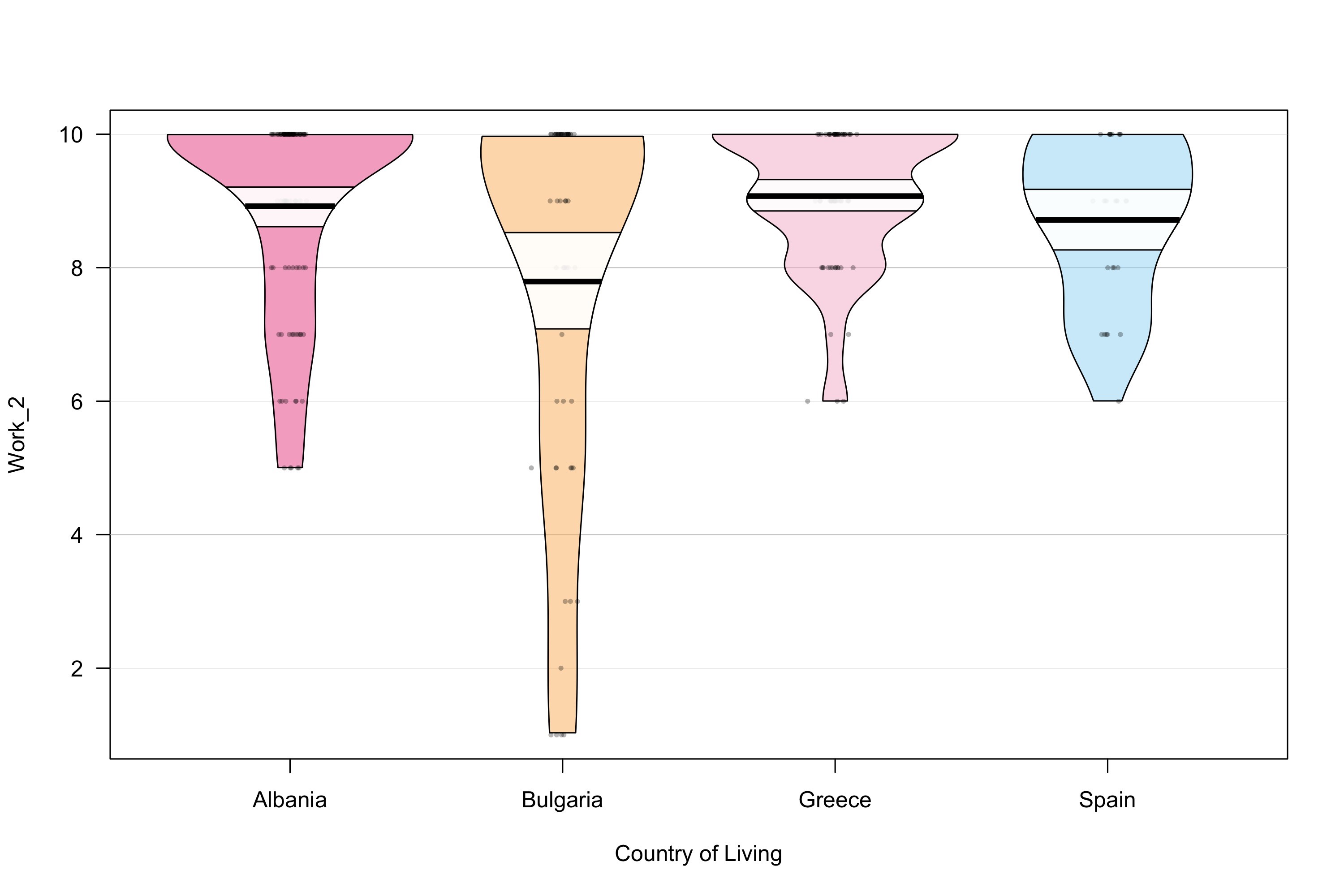 | 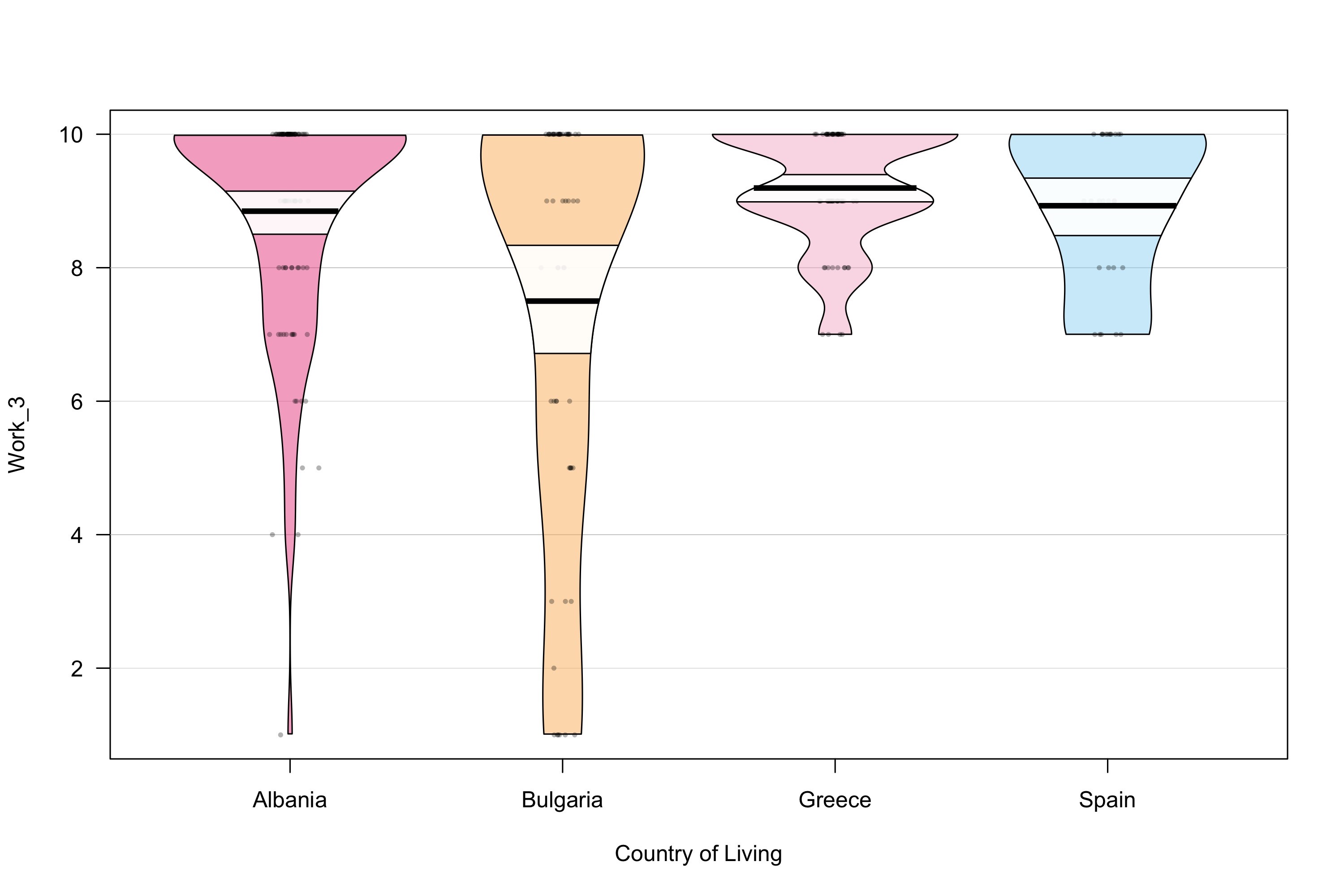 | 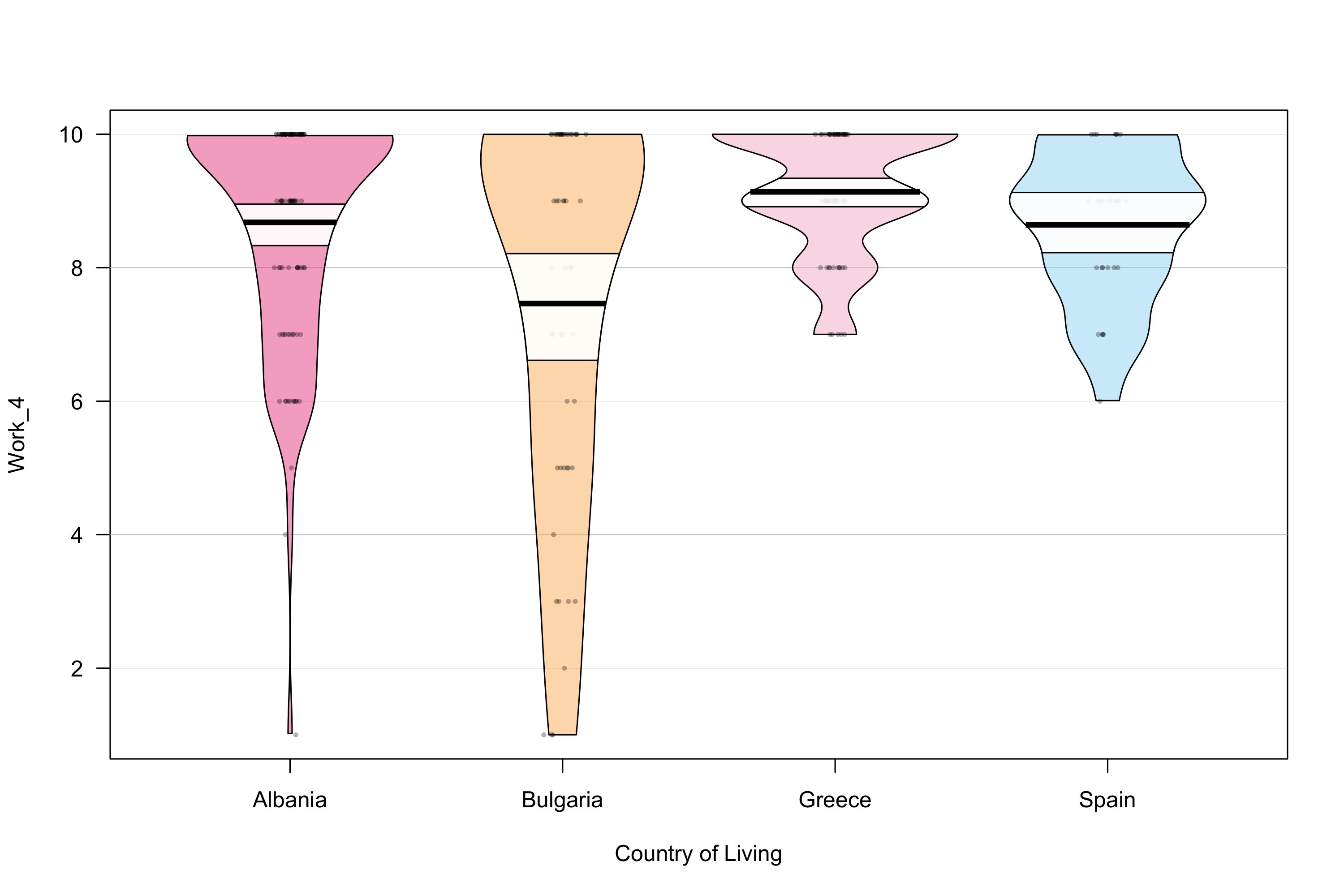 | 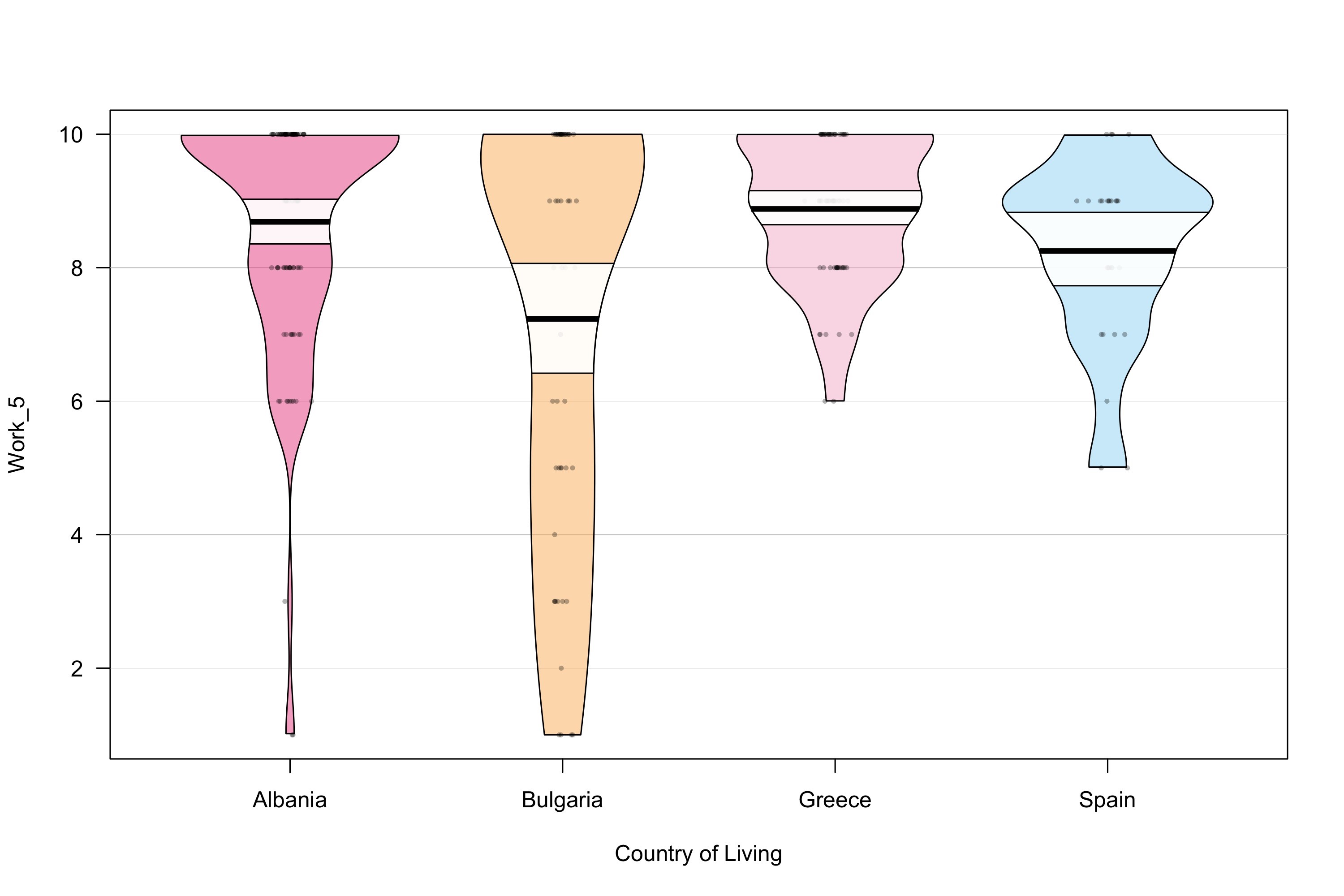 |
| --- | --- | --- | --- | --- |
| 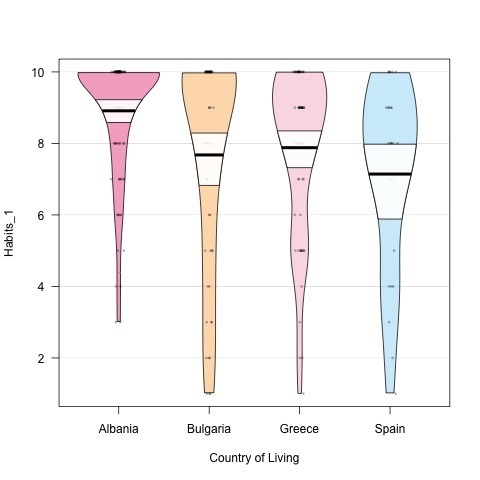 | 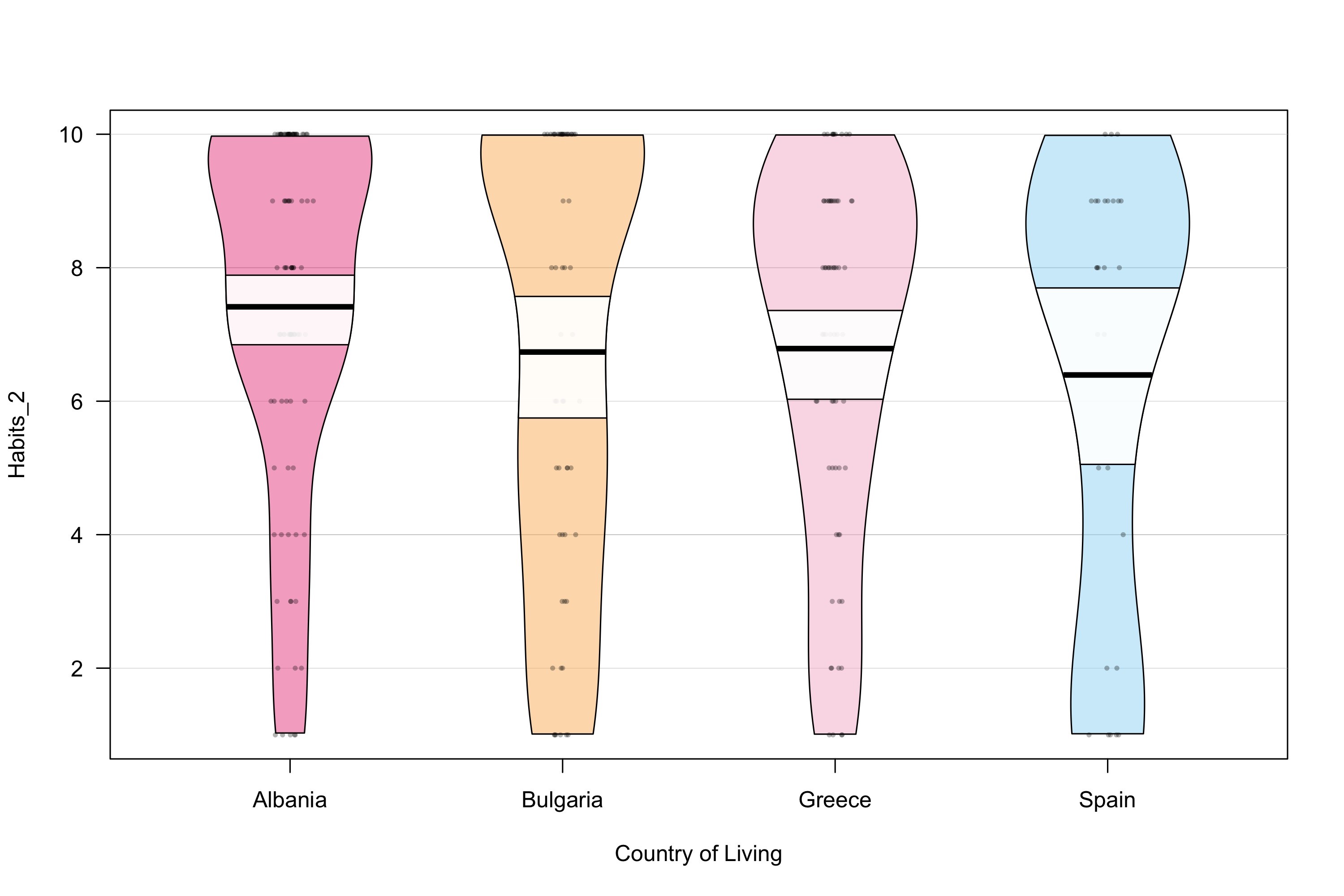 | 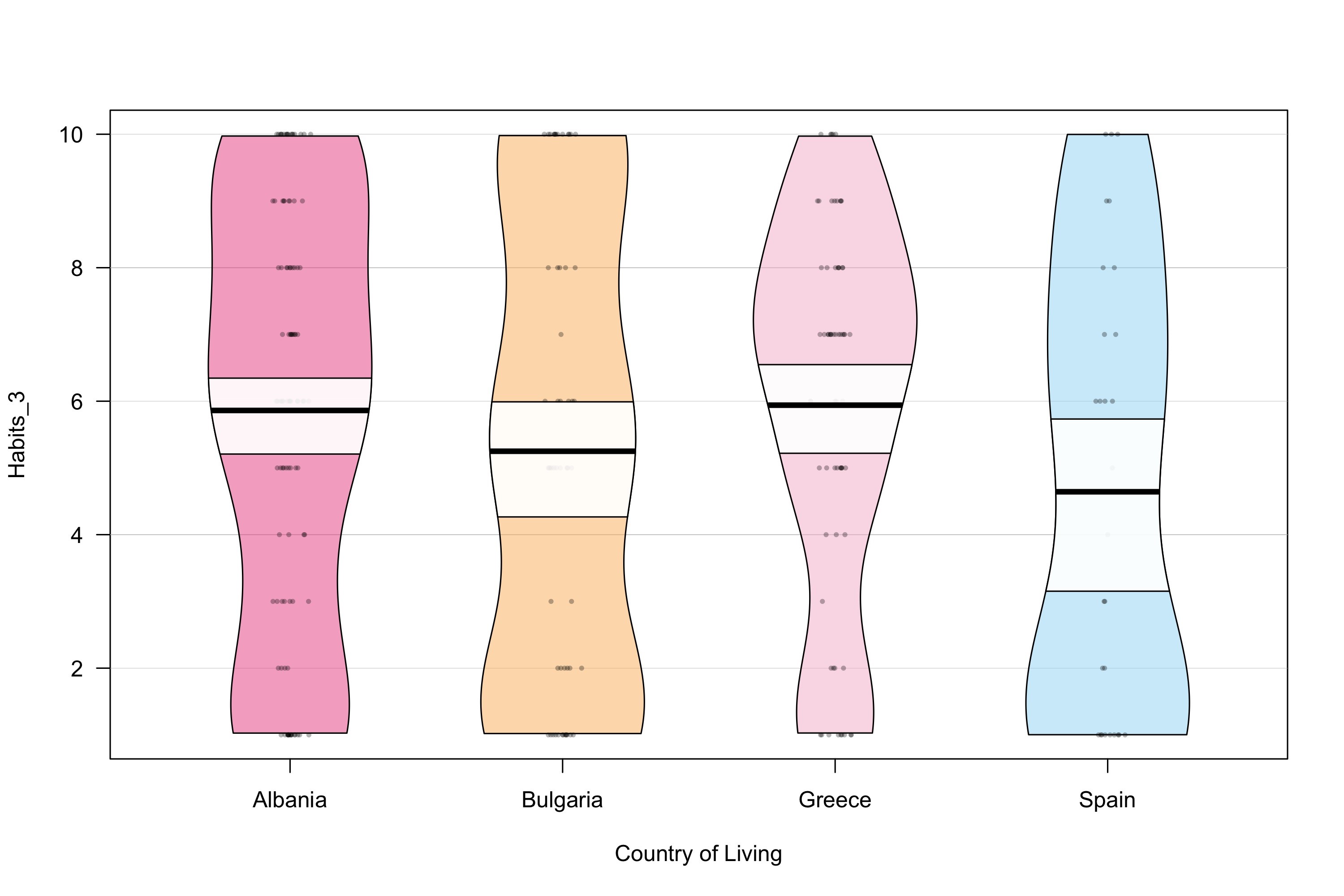 | 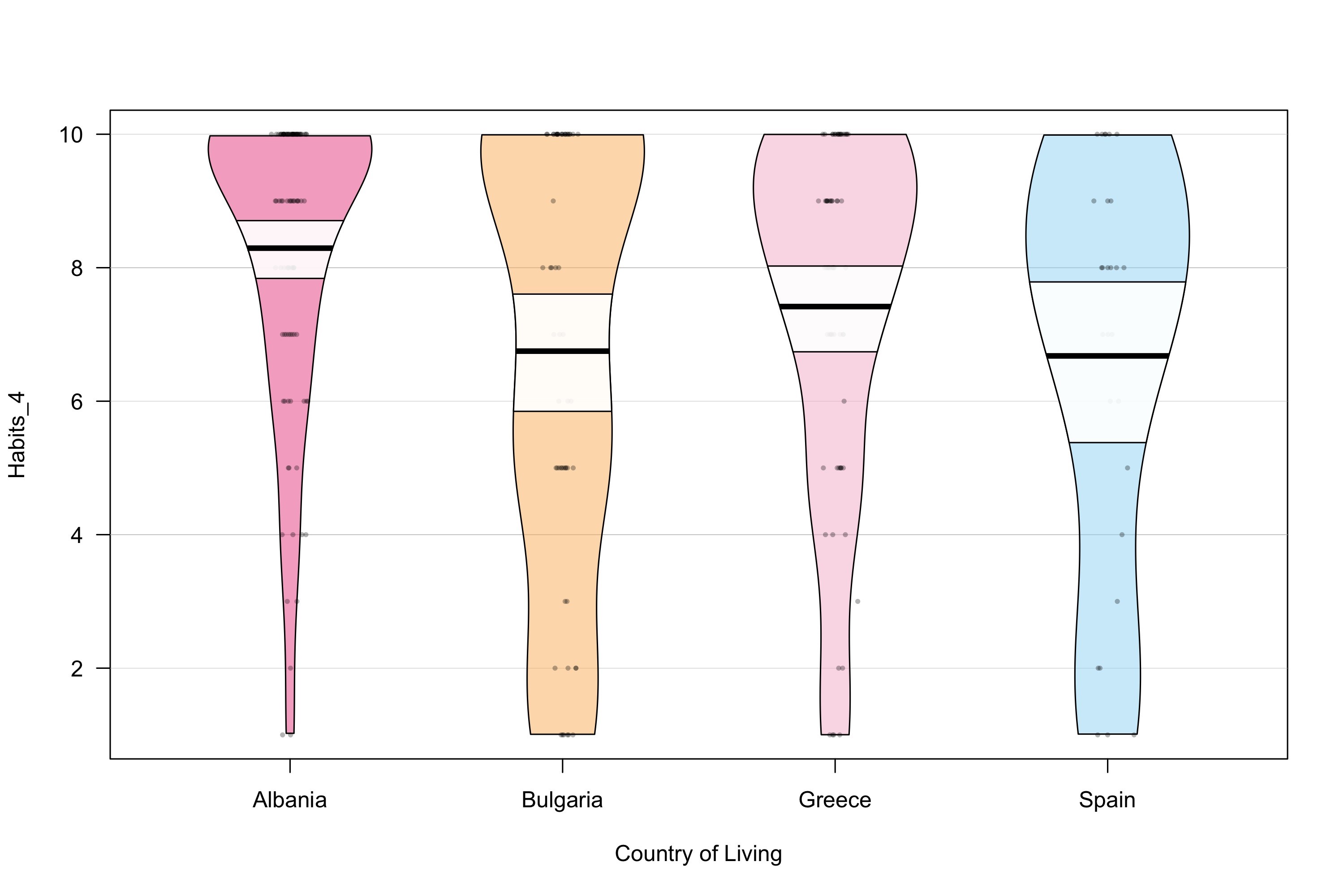 | 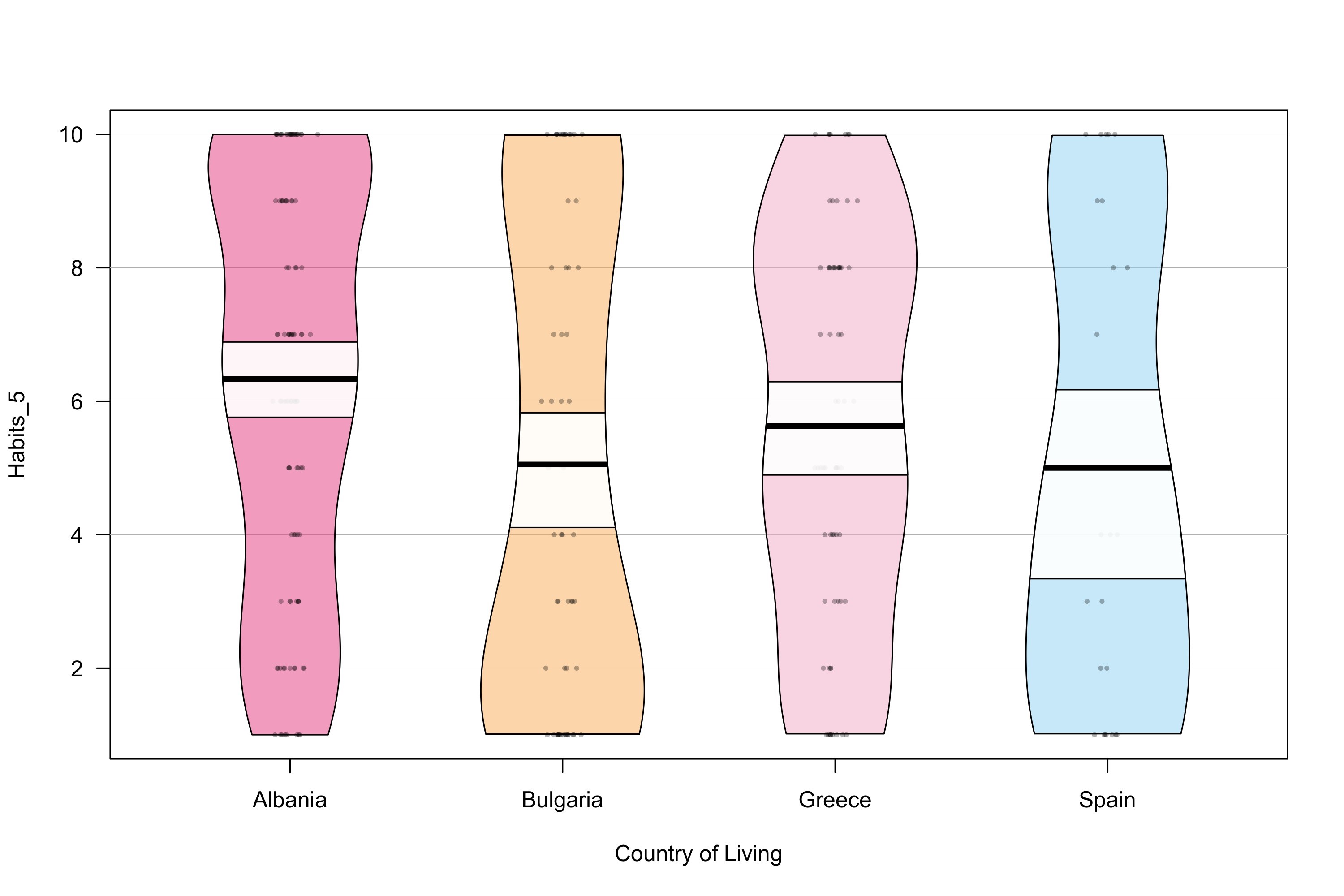 |

The table presented below displays both the mean and standard deviation of ten scores assessing individual attitudes toward technology. Specifically, five scores pertain to the use of technology in professional life, while the remaining five are associated with the utilization of technology in managing lifestyle habits. According to Table 2, the mean score is notably higher in statements related to working life. This suggests that respondents are more inclined to utilize technological devices in their professional activities compared to the management of their personal habits.

Table 2: Individual attitude towards technology: mean and standard deviation of the ten scores, across countries

|  | Albania | | Bulgaria | | Greece | | Spain | |
| --- | --- | --- | --- | --- | --- | --- | --- | --- |
|  | Mean | Std Dev. | Mean | Std Dev. | Mean | Std Dev. | Mean | Std Dev. |
| Work 1 | 8.77 | 1.79 | 7.23 | 3.32 | 9.00 | 1.28 | 8.25 | 1.35 |
| Work 2 | 8.92 | 1.55 | 7.79 | 2.93 | 9.07 | 1.07 | 8.71 | 1.21 |
| Work 3 | 8.85 | 1.71 | 7.50 | 3.15 | 9.19 | 0.89 | 8.93 | 1.15 |
| Work 4 | 8.68 | 1.69 | 7.46 | 2.89 | 9.14 | 0.94 | 8.64 | 1.13 |
| Work 5 | 8.69 | 1.85 | 7.23 | 3.10 | 8.88 | 1.08 | 8.25 | 1.38 |
| Habits 1 | 8.91 | 1.71 | 7.68 | 2.93 | 7.88 | 2.29 | 7.14 | 2.58 |
| Habits 2 | 7.41 | 2.69 | 6.74 | 3.29 | 6.79 | 2.76 | 6.39 | 3.34 |
| Habits 3 | 5.86 | 3.13 | 5.25 | 3.43 | 5.94 | 2.80 | 4.64 | 3.36 |
| Habits 4 | 8.29 | 2.26 | 6.75 | 3.29 | 7.42 | 2.69 | 6.68 | 3.09 |
| Habits 5 | 6.33 | 3.07 | 5.05 | 3.58 | 5.63 | 2.94 | 5.00 | 3.44 |

## GRM Estimation

In a GRM, each item is modeled with its own discrimination parameter and specific thresholds, corresponding to difficulty parameters that identify the boundaries between ordered response categories. Formally, a GRM employs a *I* items (*i* : 1*, . . . , I*), each with *K* response categories (*k* : 1*, . . . , K*), and models the probability of observing a response *R* greater than or equal to *k* for item *i* and individual *j*(*j* : 1*, . . . , N* ), where *N* is the sample size. This probability is defined as:

$$P\left( R_{ij}>k \right|\theta_{j} )= \frac{\exp(a_{i}(\theta_{j}-b_{ik}))}{1+ \exp(a_{i}(\theta_{j}-b_{ik}))}$$

where *a_i_* represents the discrimination parameter of item *i*, *b_ik_* is the k-th threshold for item *i*, *θ_j_* is the value of the latent trait for person *j*. The threshold *b_ik_* can be viewed as the difficulty in responding to a category equal to or greater than *k* for item *i*. In particular, the difficulty parameters signify the point at which an individual *j* with a latent trait value $\theta_{j}=b_{ik}$ has a 50% chance of responding to category *k* or higher for item *i*. Meanwhile, the *a_i_* parameters provide insights into the correlation between the latent trait and the likelihood of responding to a category higher than *k*. Naturally, the GRM is defined in terms of cumulative probabilities, but it is possible to calculate the probability of exactly observing result k using the formula:

*P* (*Y_ij_* = *k|θ_j_*) = *P* (*Y_ij_ > k* + 1*|θ_j_*) *− P* (*Y_ij_ > k|θ_j_*)

## Expanded IRT results

Here, we provide some intuition about the GRMs results. Note that following standardized guidelines about the IRT models fitting, we decide not to include the item Work 1 in the GRM model for $\hat{\theta_{w}}$ as it heavily compromises the model’s performance. Consequently, the latent trait representing the HWs’ propensity to incorporate technological devices in working activities is driven by four items. The two GRMs result to have a very good fit in the data: coefficients are statistically significant, items all highly contribute in estimating the latent trait, all the statistics and tests evaluating the overall performance of the models suggest that the models accurately fit the data and accurately predicts the latent traits (see below the complete results).

The main aspect to be evaluated when fitting an IRT model, is the contribution of the items in the models. In the presence of graded items, an item’s contribution to information is strictly related to its slope (discriminantion) parameter; the higher is the estimated parameter, the more information the item provides to the latent trait. Additionally, the more separated are the location (difficulties) parameters, the more informative is the item. Generally, an optimally informative polytomous item will exhibit a substantial location and comprehensive category coverage (as reflected by location parameters) across the latent trait’s range. We evaluate the degree of ’informativeness’ of items according to two slightly different perspectives. First, we examine the probabilities of responding to specific categories in an item’s response scale. These probabilities are graphically represented in the Category Response Curves (CRCs) presented in Figure 6. In the CRCs, discriminating items present curves that are peaked and spread over the domain of the latent trait. Figure 1 shows that the items Work 2 and Work 3 are those with the highest slopes among the items related to the usage of technology in working activities but Work 5 is also important as its estimated difficulties are well separated. Similarly, in the GRM targeted to the attitude towards technology in the management of lifestyle habits, the item Habits 2 is the one that discriminates the most, but the items Habits 1 and Habits 5 show well differentiated categories.


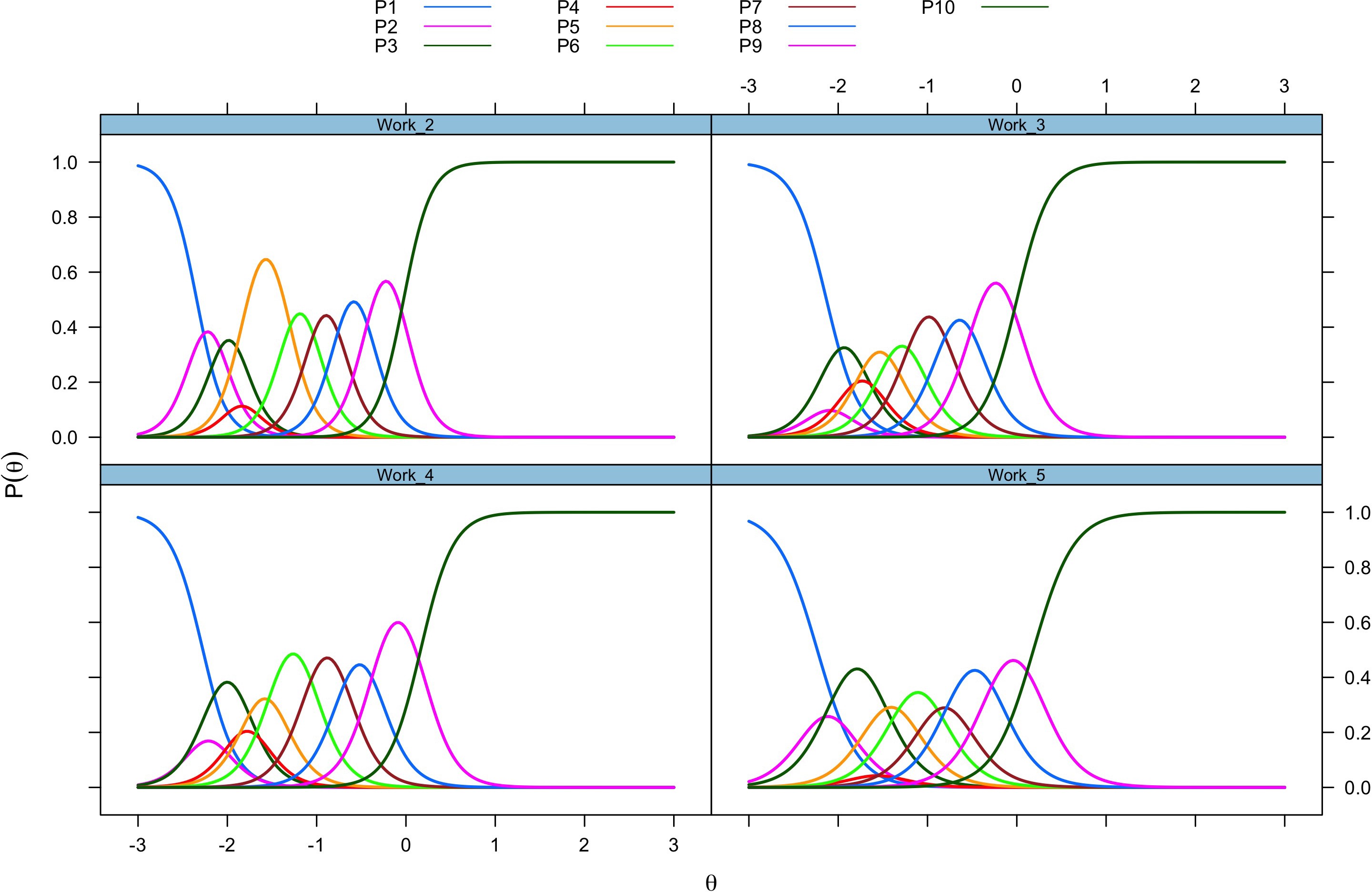

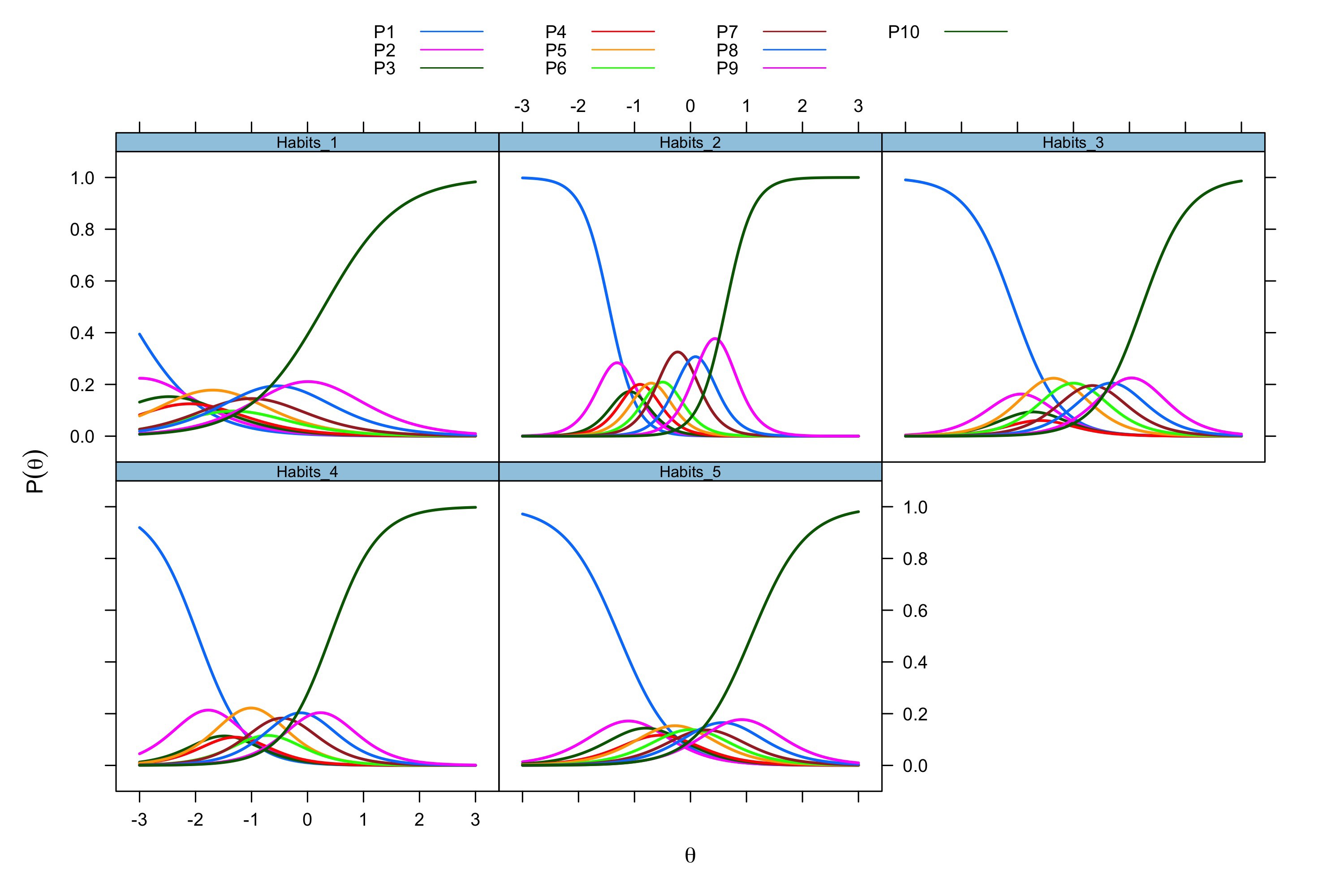


(a) Working activities (b) Everyday habits

Figure 1: Category Response Curves

Second, we assess the extent to which each item influences the overall delineation of the latent trait and in which sections of the support of *θ* its influence is mostly significant. To assess this aspect, we estimate an *Item Information Curve* (IIC) for each item that varies with *θ*, representing the share of information each item contributes to defining the latent trait. Figure 2 suggests that items mainly contribute in estimating the low-middle section of the support of the latent traits. In the model related to the usage of technologies in the working activities the item Work 1 is the most informative, while Work 5 seems to be the weakest factor. Conversely, in the model for the usage of technologies in the management of daily habits there are some items who are of paramount importance in explaining central values of the latent trait (Habits 2), and some others that result to be highly informative in predicting values located at the lower (items Habits 1 and Habits 4) or upper (items Habits 3 and Habits 5) extremes of the latent trait.


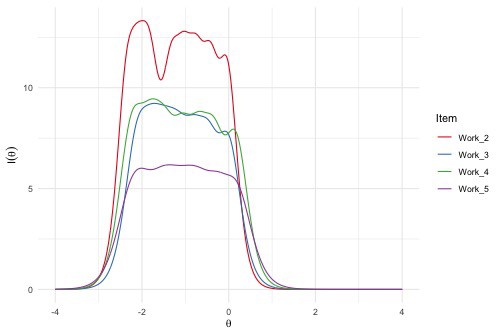

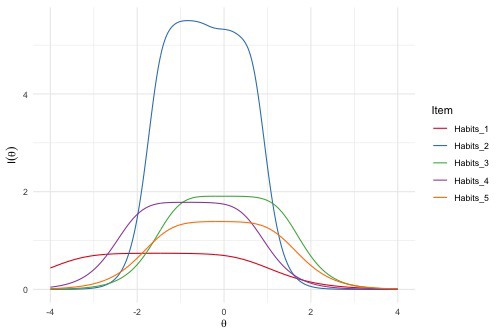


(a) Working activities (b) Everyday habits

Figure 2: Item Information Curves

Figure 3 provides the joint distribution of estimated latent traits where points are colored with respect to i) the number of missing entries in the items in the original data (left side figure) and ii) the country of living of respondents (right side figure). This kind of representation enables us to assess whether there are visible patterns in the data. In the left side Figure, there are not meaningful patterns, signaling that the joint distribution of the predicted latent traits does not vary with respect to the original presence of missing data in the items. In the right-side Figure, we can note that Bulgarian health workers tend to be grouped in the bottom left quadrant signaling lower values of the two traits. Albanian HWs are mainly located in the top right quadrant meaning that they have high values of both predicted traits; Spanish and Greece health workers represent mixed pro les, where Spanish respondents tend to have a higher attitude towards technology in working activities than their counterparts, and - vice versa - Greek respondents exhibit an higher average attitude towards the usage of technological devices in the management of their lifestyle habits, than Spanish ones.


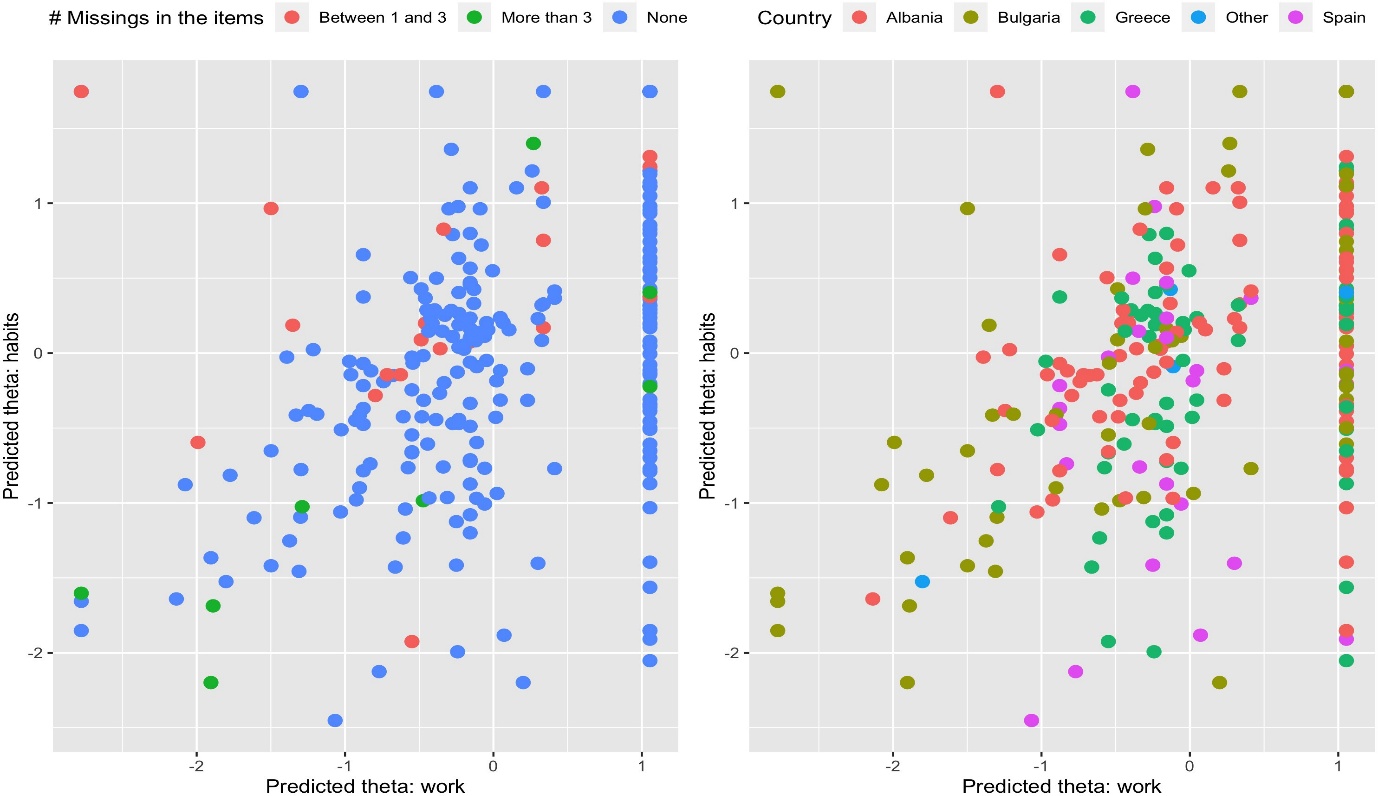


Figure 3: Joint distribution of estimated thetas

Finally, Figure 4 shows a tridimensional representation of the two estimated latent and the overall score in the items. Observations are colored with respect to the country of living.


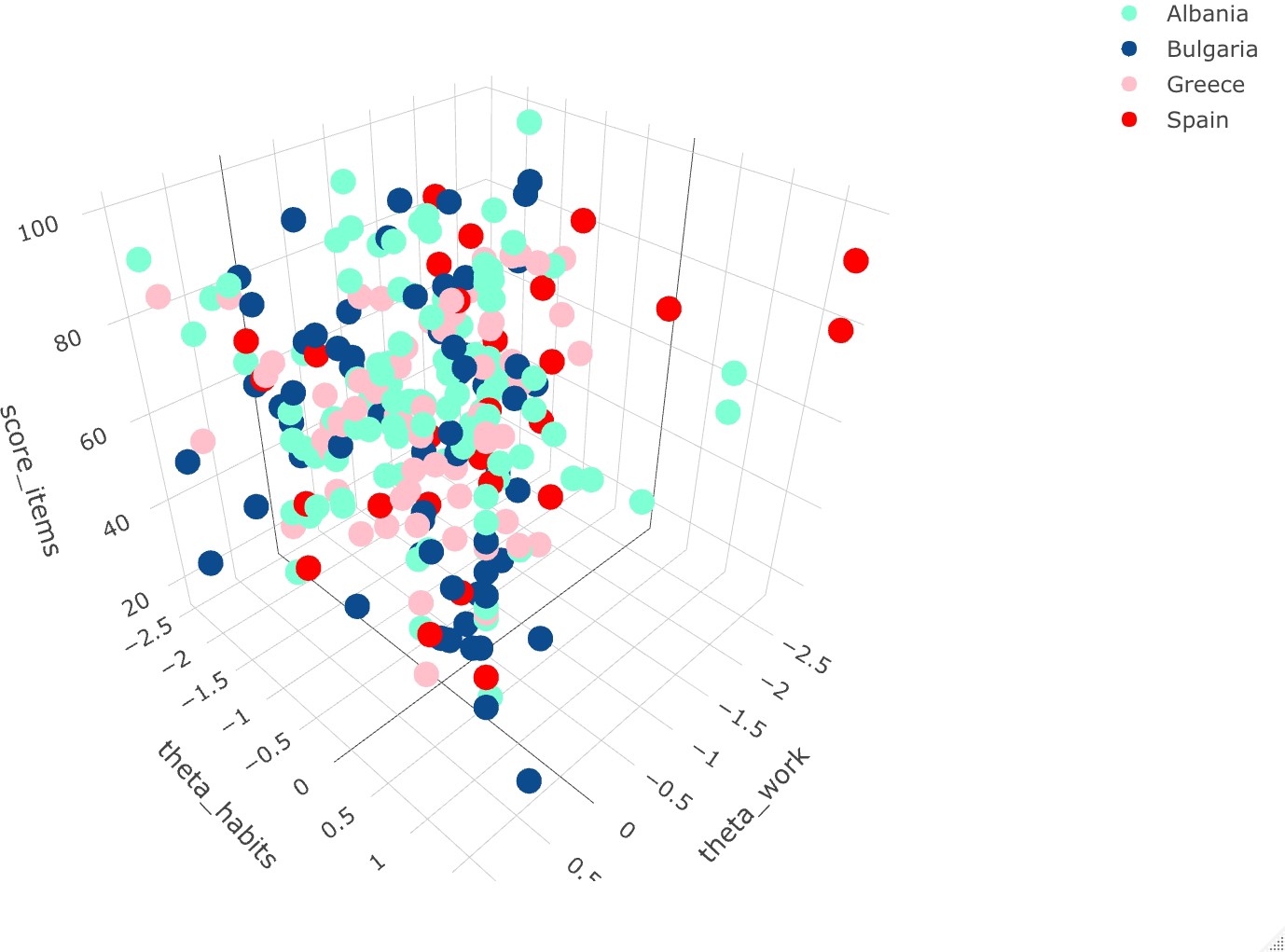


Figure 4: 3d plot of the two estimated thetas and the observed score in the items, over countries

This Section provides detailed results about the IRT models to separately estimate the latent traits *θ*_d_**_W_** and *θ*_c_**_H_**. The overall performance of the IRT models is evaluated by looking at some item fit and person-fit indices: hence, we are able to assess how well each items fits the model and how well the model is able to explain the individual responses.

### Working Activities

First, we explore the item fit. We can evaluate the contribution of items in explaining the variable within the latent trait. Table 3 provides information about the factor loadings (F1) and communalities (h2), that represent the squared factor loadings and are interpreted as the proportion of variance explained by the underlying trait in each item. In this scenario, all items exhibit a significant association (loadings GT 0.50) with the latent trait.

Table 3: IRT model for
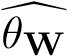
: loadings

|  | F1 | h2 |
| --- | --- | --- |
| Work 2 | 0.968 | 0.937 |
| Work 3 | 0.953 | 0.909 |
| Work 4 | 0.955 | 0.911 |
| Work 5 | 0.933 | 0.871 |

Moreover, Table 4 reports all the estimated parameters and suggests that almost all parameters are highly significant.

Table 4: IRT model for
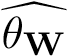
: estimated coefficients

|  | a | b1 | b2 | b3 | b4 | b5 | b6 | b7 | b8 | b9 |
| --- | --- | --- | --- | --- | --- | --- | --- | --- | --- | --- |
| Work 2 | 6.55 | -2.34 | -2.10 | -1.87 | -1.80 | -1.33 | -1.04 | -0.75 | -0.42 | -0.03 |
| CI 2.5 | 4.77 | -2.76 | -2.44 | -2.17 | -2.09 | -1.56 | -1.23 | -0.92 | -0.57 | -0.18 |
| CI 97.5 | 8.34 | -1.93 | -1.75 | -1.57 | -1.51 | -1.11 | -0.85 | -0.58 | -0.27 | 0.12 |
| Work 3 | 5.37 | -2.13 | -2.06 | -1.81 | -1.65 | -1.41 | -1.16 | -0.81 | -0.47 | 0.00 |
| CI 2.5.1 | 4.12 | -2.49 | -2.40 | -2.10 | -1.92 | -1.65 | -1.37 | -0.99 | -0.63 | -0.15 |
| CI 97.5.1 | 6.63 | -1.78 | -1.72 | -1.51 | -1.38 | -1.18 | -0.95 | -0.63 | -0.32 | 0.16 |
| Work 4 | 5.46 | -2.27 | -2.15 | -1.85 | -1.70 | -1.46 | -1.07 | -0.69 | -0.34 | 0.16 |
| CI 2.5.2 | 4.20 | -2.67 | -2.51 | -2.16 | -1.98 | -1.70 | -1.27 | -0.86 | -0.49 | 0.00 |
| CI 97.5.2 | 6.71 | -1.88 | -1.78 | -1.55 | -1.43 | -1.22 | -0.87 | -0.53 | -0.19 | 0.33 |
| Work 5 | 4.43 | -2.23 | -2.00 | -1.58 | -1.54 | -1.27 | -0.94 | -0.68 | -0.26 | 0.19 |
| CI 2.5.3 | 3.49 | -2.62 | -2.33 | -1.85 | -1.81 | -1.50 | -1.14 | -0.85 | -0.42 | 0.02 |
| CI 97.5.3 | 5.36 | -1.85 | -1.66 | -1.31 | -1.28 | -1.04 | -0.75 | -0.50 | -0.11 | 0.35 |

We can also evaluate the reliability of the items by inspecting the infit and outfit statistics. Table 5 reports both mean-squared and standardized versions of these measures: the non-standardized values should be ideally between 0.5 and 1.5.

Table 5: IRT model for *θ*_d_**_W_**: infit and outfit of the items

| item | outfit | z.outfit | infit | z.infit |
| --- | --- | --- | --- | --- |
| Work 2 | 0.46 | -1.00 | 0.78 | -1.64 |
| Work 3 | 1.80 | 1.02 | 1.20 | 1.22 |
| Work 4 | 0.83 | -0.13 | 1.00 | 0.06 |
| Work 5 | 0.70 | -0.65 | 0.89 | -0.68 |

Hence, we have good insights on the overall performance of the items in explaining the latent trait.

Second, we analyze the person fit. We can technically produce the exact same measures for each person to assess how well each person response patterns aligns with this model. The idea is that there should be relatively few people with high (low) values of the latent trait who provide low (high) answers to low difficulty (high difficulty) items: hence, the number of non - fitting respondents should be low. Figure 5 illustrate the person infit and outfit statistics.


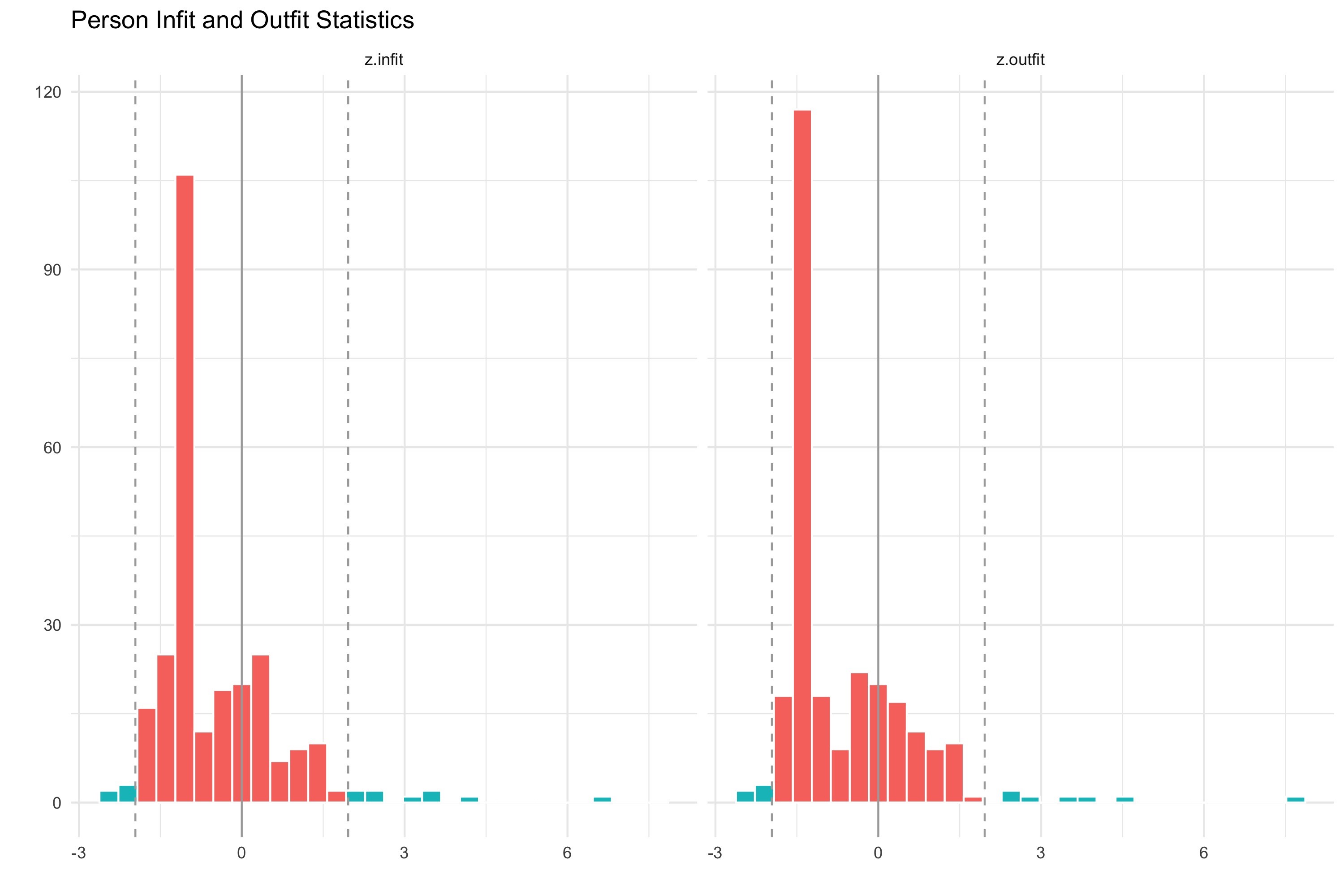


Figure 5: IRT model for
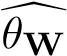
: person fit

Table 6 reports the proportion of fitting and non fitting respondents: since the proportions of non - fitting respondents is lower than 0.5, we can state that the models accurately explains people’s responses.

Table 6: IRT model for
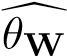
: person fit

|  | infit.outside | outfit.outside |
| --- | --- | --- |
| Prop. Fitting people | 0.95 | 0.95 |
| Prop. Non fitting people | 0.05 | 0.05 |

Finally, to evaluate the overall fit of the model we can have a look at how much information the model is able to explain on the overall domain of the latent trait. Figure 6 suggests that the algorithm very accurately predicts low-medium values of *θ*_d_**_W_**, while it is less precise in predicting high values.


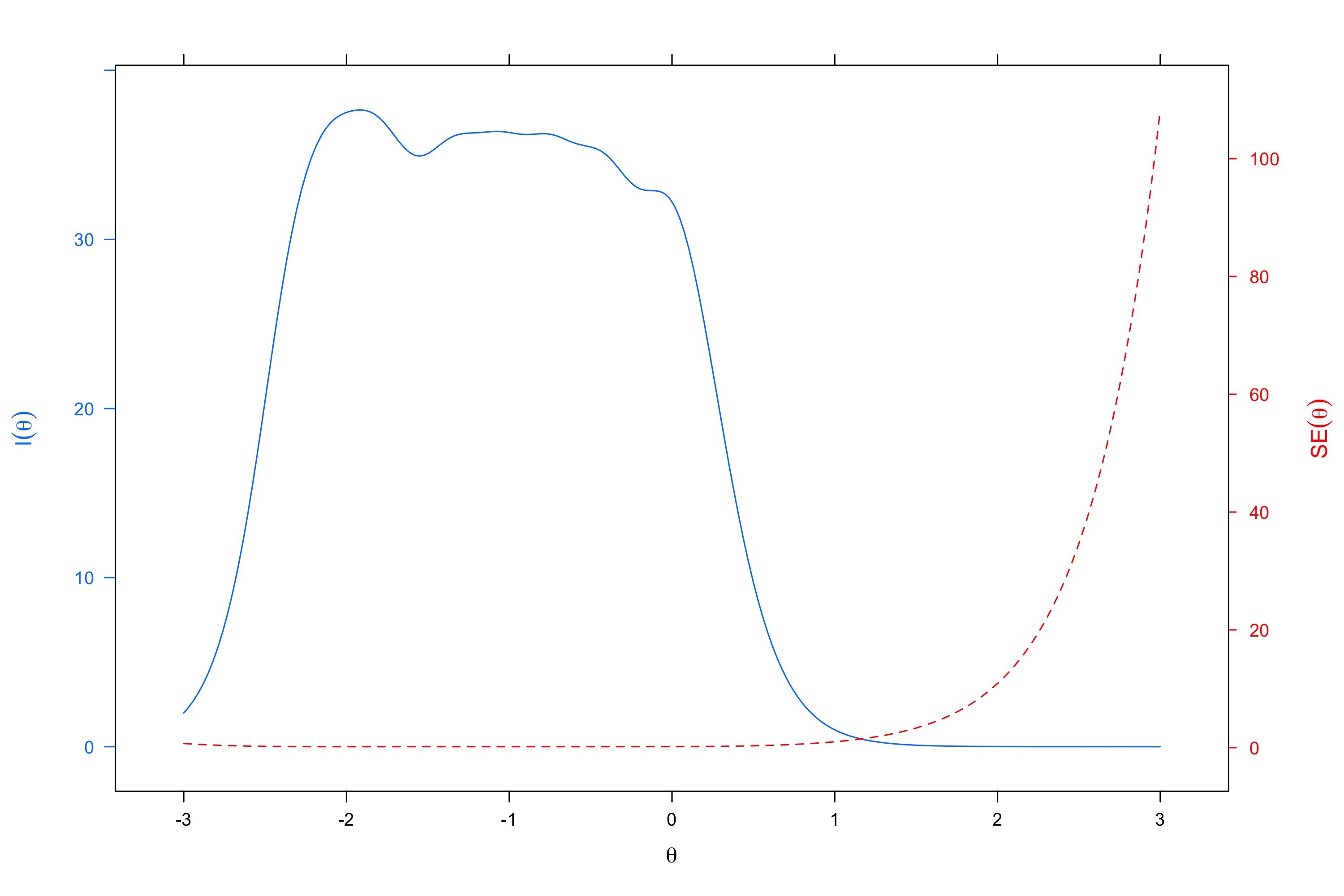


Figure 6: IRT model for
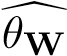
: information and standard error

Overall, the estimated reliability of the model is 0.807, which is a very good result.

### Habits

We can follow the same strategy to evaluate the performance of the IRT model to predict
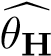
. First, we evaluate the item fit. Table 7 suggests that all items but Habits 1 contribute in explaining a relevant quote of the variance of the latent trait.

Table 7: IRT model for
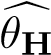
: loadings

|  | F1 | h2 |
| --- | --- | --- |
| Habits 1 | 0.661 | 0.437 |
| Habits 2 | 0.924 | 0.854 |
| Habits 3 | 0.818 | 0.669 |
| Habits 4 | 0.808 | 0.653 |
| Habits 5 | 0.770 | 0.593 |

Table 8 reports thee estimated coefficients and their corresponding confidence intervals: results suggest that all coefficients are statistically significant.

Table 8: IRT model for
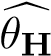
: estimated coefficients

|  | a | b1 | b2 | b3 | b4 | b5 | b6 | b7 | b8 | b9 |
| --- | --- | --- | --- | --- | --- | --- | --- | --- | --- | --- |
| Habits 1 | 1.50 | -3.29 | -2.68 | -2.27 | -1.93 | -1.45 | -1.19 | -0.80 | -0.28 | 0.29 |
| CI 2.5 | 1.11 | -4.16 | -3.34 | -2.81 | -2.40 | -1.83 | -1.53 | -1.08 | -0.51 | 0.06 |
| CI 97.5 | 1.89 | -2.41 | -2.02 | -1.72 | -1.47 | -1.08 | -0.86 | -0.53 | -0.05 | 0.52 |
| Habits 2 | 4.11 | -1.45 | -1.17 | -1.00 | -0.80 | -0.60 | -0.39 | -0.06 | 0.24 | 0.63 |
| CI 2.5.1 | 2.91 | -1.71 | -1.39 | -1.20 | -0.99 | -0.77 | -0.56 | -0.22 | 0.08 | 0.45 |
| CI 97.5.1 | 5.31 | -1.20 | -0.95 | -0.80 | -0.62 | -0.43 | -0.23 | 0.09 | 0.40 | 0.81 |
| Habits 3 | 2.42 | -1.07 | -0.80 | -0.65 | -0.54 | -0.17 | 0.18 | 0.50 | 0.85 | 1.23 |
| CI 2.5.2 | 1.88 | -1.31 | -1.01 | -0.84 | -0.74 | -0.35 | -0.01 | 0.31 | 0.62 | 0.96 |
| CI 97.5.2 | 2.96 | -0.83 | -0.59 | -0.45 | -0.35 | 0.01 | 0.36 | 0.70 | 1.07 | 1.49 |
| Habits 4 | 2.33 | -1.96 | -1.59 | -1.39 | -1.20 | -0.81 | -0.62 | -0.30 | 0.05 | 0.41 |
| CI 2.5.3 | 1.80 | -2.33 | -1.90 | -1.68 | -1.46 | -1.04 | -0.82 | -0.48 | -0.13 | 0.21 |
| CI 97.5.3 | 2.86 | -1.58 | -1.27 | -1.10 | -0.94 | -0.59 | -0.41 | -0.11 | 0.24 | 0.61 |
| Habits 5 | 2.05 | -1.28 | -0.94 | -0.65 | -0.43 | -0.13 | 0.15 | 0.41 | 0.74 | 1.09 |
| CI 2.5.4 | 1.60 | -1.56 | -1.18 | -0.87 | -0.63 | -0.32 | -0.05 | 0.21 | 0.51 | 0.82 |
| CI 97.5.4 | 2.50 | -0.99 | -0.70 | -0.44 | -0.23 | 0.06 | 0.34 | 0.62 | 0.97 | 1.35 |

Looking at the infit and outfit statistics (Table 9) we see that they are all between 0.5 and 1.5, meaning that items play a positive role in estimating the latent trait.

Table 9: IRT model for
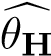
: infit and outfit of the items

| item | outfit | z.outfit | infit | z.infit |
| --- | --- | --- | --- | --- |
| Habits 1 | 0.69 | -1.87 | 0.88 | -1.05 |
| Habits 2 | 0.44 | -1.70 | 0.56 | -4.29 |
| Habits 3 | 0.84 | -1.14 | 0.89 | -1.11 |
| Habits 4 | 0.67 | -1.54 | 0.81 | -1.85 |
| Habits 5 | 0.89 | -0.88 | 0.92 | -0.90 |

Hence, as before, the items appears to be highly informative in predicting the latent trait. Figure 6 illustrate the person infit and outfit statistics.


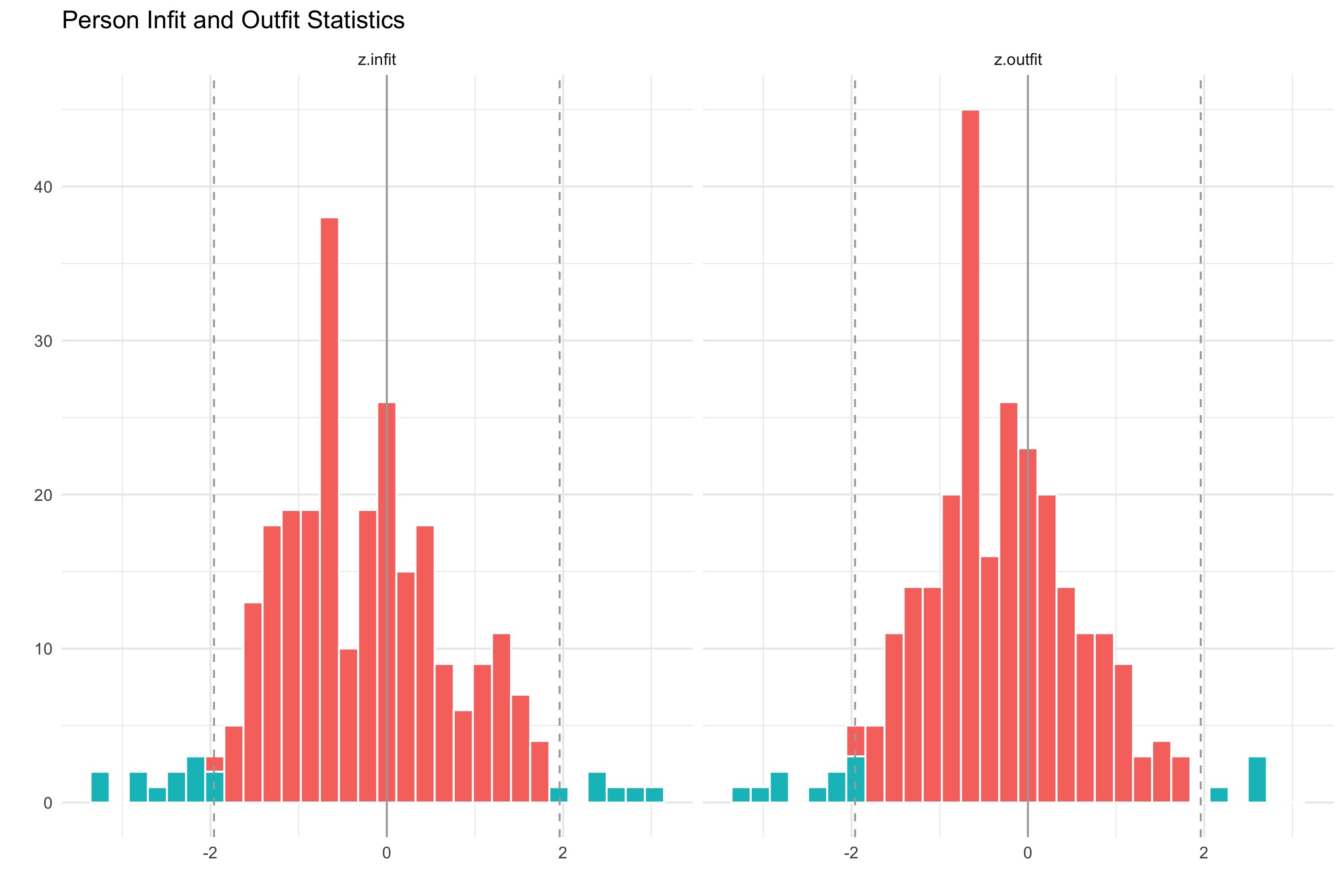


Figure 6: IRT model for
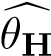
: person fit

Table 10 reports the proportion of fitting and non fitting respondents: these proportions are very low (even if the one regarding the infit respondents is slightly above 0.05) and therefore we are inclined to conclude that the model has an adequate person fit.

Table 10: IRT model for
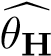
: person fit

|  | infit.outside | outfit.outside |
| --- | --- | --- |
| Prop. Fitting people | 0.93 | 0.95 |
| Prop. Non fitting people | 0.07 | 0.05 |

Finally, to evaluate the overall fit of the model we can have a look at how much information the model is able to explain on the overall domain of the latent trait. Figure 7 suggests that the algorithm very accurately predicts central values of
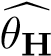
, while it is less precise in predicting both extremely low values and extremely high values. the estimated reliability of the model is 0.88, which is a very good result.


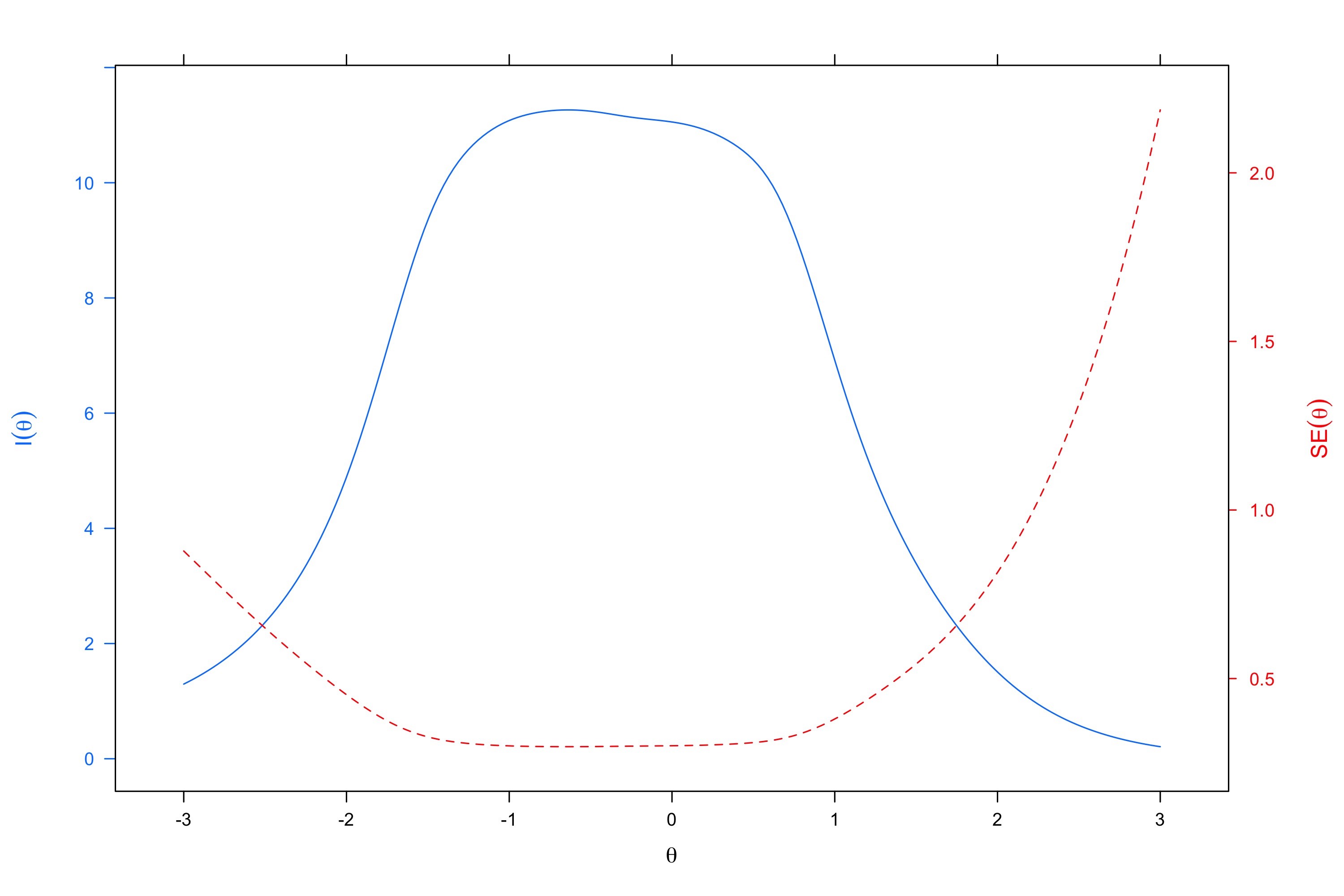


Figure 7: IRT model for
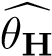
: information and standard error Overall,

## The determinants of the individual attitude towards technology

Table 11: The determinants of the individual attitude towards technology: regressions

|  | Theta work | Theta habits Theta whole Score items | | |
| --- | --- | --- | --- | --- |
|  | 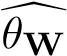 | 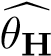 | $\hat{\theta}_{WH}$ |  |
| Gender: Male | −0.25^∗^ | −0.18 | −0.44^∗^ | −4.44^∗^ |
|  | (0.14) | (0.14) | (0.23) | (2.57) |
| Gender: Prefer not to answer | 0.79 | 0.89 | 1.68 | 19.80 |
|  | (0.65) | (0.64) | (1.06) | (12.00) |
| Country: Bulgaria | −0.42^∗∗∗^ | −0.33^∗∗^ | −0.75^∗∗∗^ | −12.70^∗∗∗^ |
|  | (0.16) | (0.15) | (0.25) | (2.87) |
| Country: Greece | 0.03 | −0.45^∗∗∗^ | −0.42 | −3.81 |
|  | (0.16) | (0.15) | (0.26) | (2.90) |
| Country: Other | −0.04 | −0.20 | −0.23 | −4.29 |
|  | (0.43) | (0.42) | (0.70) | (7.92) |
| Country: Spain | −0.25 | −0.60^∗∗∗^ | −0.85^∗∗^ | −9.52^∗∗^ |
|  | (0.22) | (0.21) | (0.35) | (3.99) |
| Age: Under 30 | 0.20 | 0.02 | 0.22 | 3.20 |
|  | (0.12) | (0.12) | (0.20) | (2.26) |
| Education: High Educ | 0.30^∗^ | 0.48^∗∗∗^ | 0.78^∗∗∗^ | 10.30^∗∗∗^ |
|  | (0.17) | (0.17) | (0.29) | (3.23) |
| Usage EHR: EHR yes | 0.02 | −0.01 | 0.02 | 1.25 |
|  | (0.13) | (0.13) | (0.21) | (2.36) |
| Doctor | −0.09 | −0.48^∗∗∗^ | −0.57^∗∗∗^ | −6.58^∗∗∗^ |
|  | (0.13) | (0.13) | (0.21) | (2.38) |
| Constant | −0.12 | 0.17 | 0.05 | 75.60^∗∗∗^ |
|  | (0.22) | (0.22) | (0.36) | (4.08) |
| *N* | 265 | 265 | 265 | 265 |
| R2 | 0.09 | 0.13 | 0.13 | 0.18 |
| Adjusted R^2^ | 0.06 | 0.10 | 0.10 | 0.15 |
| Residual Std. Error (df = 254) | 0.91 | 0.89 | 1.48 | 16.70 |
| F Statistic (df = 10; 254) | 2.64^∗∗∗^ | 3.80^∗∗∗^ | 3.88^∗∗∗^ | 5.54^∗∗∗^ |
| *Notes:* |  | ^∗∗∗^Significant at the 1 percent level. ^∗∗^Significant at the 5 percent level. ^∗^Significant at the 10 percent level. | | |
