# Supplementary material for: Understanding Health Care Workers’ Attitudes and Preferences Toward Digital Patient Monitoring Platforms: Cross-Country Survey Study
Source: JMIR Form Res. 2025 Sep 23;9:e67142. doi: 10.2196/67142 (PMC12456460; doi:10.2196/67142)
Supplement: Checklist 1 [file formative-v9-e67142-s006.pdf]

**Checklist for Reporting Results of Internet E-Surveys (CHERRIES)**		
Item Category	***Checklist Item***	***Explanation***
**Design**		
	Describe survey design	This was a cross-sectional online survey administered to a convenience sample of European health workers (HWs). The survey consisted of 3 sections: Section 1 - Demographics, Section 2 - evaluates the baseline individual attitude towards technology Section 3- DCE experiment to elicit the HWs preferences. Survey questions are listed in Appendix B.
**IRB (Institutional Review Board) approval and informed consent process**		
	IRB approval	The study was carried out in compliance and accordance with the General Data Protection Regulation (2016/679) and the Italian Legislative Decree No. 196/2003 ("Personal Data Protection Code ")<br>The data used in the study are from a web survey which is completely anonymous and does not collect personally identifiable information. In particular, a link to a survey is sent to an individual e-mail address(es) and/or through QR code. Responses are submitted directly to a survey software package (not returned via email); the questionnaire does not gather any personally identifiable information for any purpose, or a combination of identifiers that may make it more likely to identify an individual, and individual respondents' responses/data cannot be linked to their email.<br>The data are anonymous and do not interfere with the rights and freedoms of the respondents; therefore, no

Checklist for Reporting Results of Internet E-Surveys (CHERRIES)		
Item Category	***Checklist Item***	***Explanation***
		evaluation by the institutional ethics committee is necessary.
	Informed consent	The survey included a disclosure describing the project and the purpose of the survey, the section of the questionnaire, the length of time of the survey (about 15 minutes), that participation and consent were totally voluntary that the questionnaire does not collect personally identifiable information and that the participants might skip questions they don't want to answer.
	Data protection	The data used in the study are from a web survey which is completely anonymous and does not collect personally identifiable information.
**Development and pre-testing**		
	Development and testing	Our initial survey design was performed within the DIGICARE consortium to design questionnaire sections and sampling methodology. Additional insights were gathered through focused group discussions involving health workers and healthcare policy makers across the four implementation countries (see Appendix A). We pilot tested the survey on the Qualtrics platform.
**Recruitment process and description of the sample having access to the questionnaire**		
	Open survey versus closed survey	This was an open survey
	Contact mode	The link to the web survey was shared by email from the healthcare professionals enrolled into the DigiCare consortium to other HWs of

Checklist for Reporting Results of Internet E-Surveys (CHERRIES)		
Item Category	*Checklist Item*	*Explanation*
		their knowledge who would potentially participate to the study. In the email potential participants were asked to further circulate the link to the survey to their colleagues so to create a kind of snowball sampling.
	Advertising the survey	The survey was advertised online, through the mailing lists including colleagues of the healthcare professionals enrolled into the DigiCare consortium.
**Survey administration**		
	Web/E-mail	Surveys were distributed via e-mail and QR-codes. Responses were collected automatically with the Qualtrics platform.
	Context	The survey did not require a website
	Mandatory/voluntary	The survey did not require mandatory participation.
	Incentives	No incentives were offered
	Time/Date	The survey was launched in April 2022 and ended in November 2023
	Randomization of items or questionnaires	The questions related to the first two sections were not randomized, while in the third section (DCE) respondents faced scenarios comparing alternative web platforms for patient data monitoring, that were randomly generated (attributes and levels of the alternative web platforms proposed in the DCE were randomly assigned to respondents according to a randomization design).
	Adaptive questioning	No.
	Number of Items	There are three or four questions per page, and each of the six DCE comparison is included in one dedicated page.

Checklist for Reporting Results of Internet E-Surveys (CHERRIES)		
Item Category	***Checklist Item***	***Explanation***
	Number of screens (pages)	Nine, but six pages are dedicated to the DCE comparisons (one page includes one comparison).
	Completeness check	Then, the dataset automatically generated by Qualtrics included a variable signaling whether the questionnaire was completed or not.
	Review step	Respondents were able to press the "Back" button, but they were not able to review their responses right before submitting the questionnaire.
**Response rates**		
	Unique site visitor	This was not applicable as we did not have a web page.
	View rate (Ratio of unique survey visitors/unique site visitors)	This was not applicable as we did not have a web page.
	Participation rate (Ratio of unique visitors who agreed to participate/unique first survey page visitors)	The final sample of responses has been obtained through a snowball sampling, where healthcare professionals were asked to circulate the web survey link to their peers and colleagues. It is not possible to derive the number of people receiving that link. So, it's not possible for us to compute any kind of participation rates.
	Completion rate (Ratio of users who finished the survey/users who agreed to participate)	The final sample of responses has been obtained through a snowball sampling, where healthcare professionals were asked to circulate the web survey link to their peers and colleagues. It is not possible to derive the number of people receiving that link. So, it's not possible for us to calculate how many people agreed to participate. However, we are able to know the percentage of people who started the survey, without actually finishing it, and it equals to 12%.

Checklist for Reporting Results of Internet E-Surveys (CHERRIES)		
Item Category	*Checklist Item*	*Explanation*
**Preventing multiple entries from the same individual**		
	Cookies used	Qualtrics automatically derives an ID identifier, without using cookies. There is also a "I'm not a robot" check at the end of the first page.
	IP check	It is not possible for a respondent to repeat the questionnaire through the same browser.
	Log file analysis	None.
	Registration	It is not possible for a respondent to repeat the questionnaire through the same browser.
**Analysis**		
	Handling of incomplete questionnaires	We analyzed all questionnaires related to respondents who went through all pages of the questionnaire (the dataset generated by Qualtrics included a variable named Progress that equals 100 if the respondent has read the whole questionnaire). Final data may include missing entries in the first two sections (section three dedicated to the DCE comparisons did not allow respondents to skip comparison) and, while we took missing information from section one as given, we decided to impute missing items in section two (they represent the 3% of all responses) using predictive mean matching to compute the IRT traits for all respondents.
	Questionnaires submitted with an atypical timestamp	Not applicable
	Statistical correction	No.
